# Using Noncovalent Interactions to Test the Precision of Projector-Augmented Wave Data Sets

**DOI:** 10.1021/acs.jctc.3c00930

**Published:** 2023-12-01

**Authors:** Sirous Yourdkhani, Jiří Klimeš

**Affiliations:** Department of Chemical Physics and Optics, Faculty of Mathematics and Physics, Charles University, Prague 2 CZ-12116, Czech Republic

## Abstract

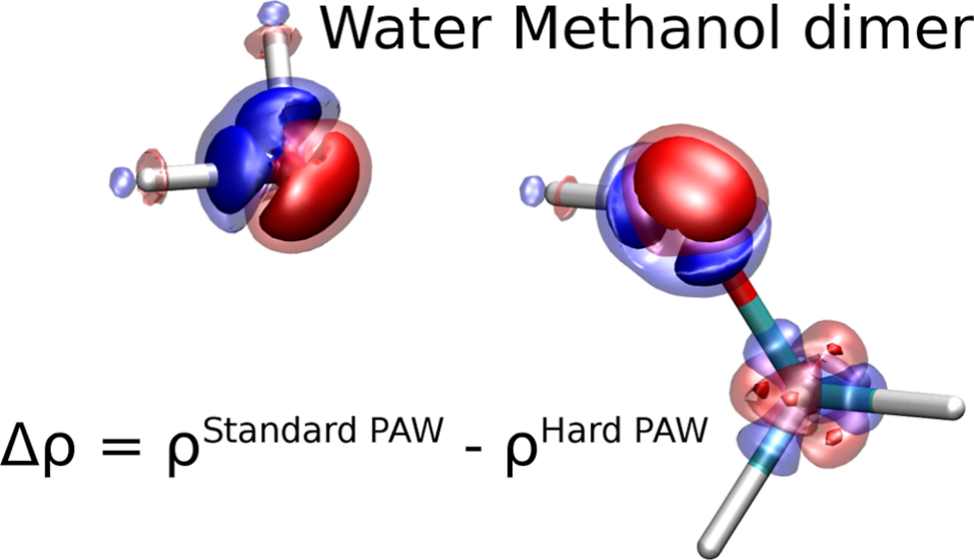

The projector-augmented wave (PAW) method is one of the approaches that are widely used to approximately treat core electrons and thus to speed up plane-wave basis set electronic structure calculations. However, PAW involves approximations, and it is thus important to understand how they affect the results. Tests of the precision of PAW data sets often use the properties of isolated atoms or atomic solids. While this is sufficient to identify problematic PAW data sets, little information has been gained to understand the origins of the errors and suggest ways to correct them. Here, we show that the interaction energies of molecular dimers are very useful not only to identify problematic PAW data sets but also to uncover the origin of the errors. Using dimers from the S22 and S66 test sets and other dimers, we find that the error in the interaction energy is composed of a short-range component with an exponential decay and a long-range electrostatic part caused by an error in the total charge density. We propose and evaluate a simple improvable scheme to correct the long-range error and find that even in its simple and readily usable form, it is able to reduce the interaction energy errors to less than half on average for hydrogen-bonded dimers.

## 1 Introduction

There are several choices that one needs to make when performing total energy calculations at the level of quantum mechanics that affect the quality of the result. We often focus on the accuracy of approximations involving Hamiltonian or wave functions such as Hartree–Fock or density functional theory (DFT). However, there are also many numerical parameters that need to be set or approximations that one might use and that affect the energy as well. When using a plane-wave (PW) basis set and periodic boundary conditions, one usually uses pseudopotentials (PP)^1−3^ or the projector-augmented wave (PAW) method.^4−7^ The use of PPs or PAW reduces the size of the PW basis set needed to describe the states, and this typically speeds up the calculations.

The PAW is an all-electron approach; however, the core and valence electrons are treated in a different way. The core electrons are usually kept frozen in orbitals obtained for isolated atoms.^8^ For valence electrons, the all-electron orbitals |ϕ_*i*_⟩ are formally written in terms of pseudostates  using a transformation . The |χ_*n*_⟩ and  are partial waves obtained for an isolated atom with all-electron potential and with pseudopotential, respectively, and |*p*_*n*_⟩ are projectors on the pseudo partial waves. The pseudopotential differs from the all-electron potential only within some cutoff radius around the nuclei, and thus, the partial waves differ also only around the nuclei. The pseudostates are optimized, and the transformation is used to derive terms in the Hamiltonian that account for all-electron potential within the cutoff radius. The cutoff radius is an important parameter that affects the precision of the result. The precision generally decreases with increasing cutoff distance, but the pseudostates are smoother, and a smaller PW cutoff can be used. Generally, the PPs or PAWs with small cut-offs are called hard, and those with larger cut-offs are soft.^9^ Often, there are several PPs or PAWs available from which one can choose depending on the required precision of the calculation and available computational time. For example, in the Vienna ab initio simulations package (VASP), one can choose from Soft, Standard, and Hard PAWs;^6^ there are harder and softer PAW potentials for some elements available in pslibrary.^10^

The precision of different PPs or PAW data sets can be assessed by comparison to all-electron results calculated, e.g., with the full potential linearized plane wave scheme (FLAPW)^11^ or using Gaussian^12^ or Slater basis sets.^13^ For example, Lejaeghere et al.^14^ used the FLAPW scheme with local orbitals to test a large number of PPs and PAW data sets for the prediction of equilibrium volume and bulk modulus of elemental solids.^15^ The so-called Δ-factor by Lejaeghere et al.,^15^ which is a measure of the error, was used in several works that developed PPs, or PAW potentials, or their libraries.^16,17^ However, assessments of the precision of PPs or PAWs for other properties that would cover the majority of elements are scarce, and more often, the information about the precision of PPs or PAWs is only a byproduct of studies that focus on other developments. In this regard, Paier et al.^18^ compared atomization energies for the G2–1 data set of Frisch et al.^100^ obtained with Perdew–Burke–Ernzerhof (PBE)^19^ and PBE0^20^ exchange–correlation (XC) functionals in the PAW formalism to the corresponding results in large Gaussian basis sets.^21,22^ Furthermore, Maggio et al.^23^ tested the PAW approximation for predicting *G*_0_*W*_0_(24) energies on the *GW*100 data set.^25^ Finally, using an all-electron Gaussian reference, Adllan and Dal Costro^26^ compared PAW and PP schemes on a set of atomization energies, bond distances, and vibrational frequencies of diatomic molecules of 13 elements. In these tests, it was noted that the agreement between PW–PAW calculations and the all-electron reference decreases for molecules containing nitrogen, oxygen, fluorine, or sulfur. This is consistent with the results obtained by Lejaeghere and co-workers,^14^ but generally, there is not much information gained than that for specific elements (oxygen and nitrogen), harder PPs or PAWs need to be used.

The binding energies of molecular dimers or molecular solids are often considerably affected by the choice of the PP or PAW data set. For example, for the α polymorph of oxalic acid, one obtains a binding energy of −1289 meV using VASP’s Standard PAWs and the PBE + vdW^TS^ method.^27^ This is several percent away from the value of −1249 meV obtained for the Hard PAWs.^28^ The discrepancy between the results of Standard and Hard PAWs can be less problematic for other systems. For example, the binding energy changes only by around 4 meV for ammonia crystals, from −459 to −463 meV. Nevertheless, these imprecisions clearly affect the results of benchmarking studies. Clearly, the interaction or binding energies are sensitive to the choice of PPs or PAWs and can thus be used to assess their precision. Such an approach was already used by Witte and co-workers,^29^ who compared the precision of norm-conserving^1^ (NC) Troullier-Martins pseudopotentials^2^ against all-electron data using PBE and Slater exchange with PW92 correlation (SPW92)^30^ functionals on the S22 database.^31^ The authors noticed that convergence of the interaction energy with the PW basis set size is slower for molecules containing oxygen or nitrogen. Moreover, Tosoni et al.^32^ compared Troullier-Martins pseudopotentials to all-electron calculations for the molecular crystal of formic acid. While the agreement between both approaches was considered good, the identified deviations could be due to both unconverged Gaussian basis sets and imprecise PPs.

In this study, we analyze the precision of the PAW potentials using the interaction energies of dimers in the S22 and S66 sets^31,33^ as well as additional dimers to assess the distance dependence of the errors. The S22 and S66 sets are targeted at biomolecules; thus, the molecules are formed by hydrogen, carbon, nitrogen, and oxygen. The sets cover a range of interactions, from those with large electrostatic contributions to those with dominant dispersion. We test various PAW potentials supplied with the Vienna ab initio simulation package (VASP)^34−37^ as well as those in pslibrary.1.0.0^10,38^ against reference interaction energies calculated using large Gaussian basis sets. We obtain data for the PBE functional, which was used to construct the PAW data sets, as well as for the HF method, which allows assessment of the transferability of the PAWs. The tests on S22 and S66 are followed by the calculation and analysis of errors for the binding curves of molecular dimers. Finally, the data show that the error in the interaction energy mostly occurs due to an incorrect description of the electron density, and we devise and test a simple correction for the error.

## 2 Methods Overview

### 2.1 Data Sets

For the tests of binding energies of dimers in the S22^31^ and S66^33^ databases, we used the structures available on the begdb.org website with no additional structure optimization.^39^ To further understand the precision of the different PAW potentials, we constructed additional dimers in which molecules were oriented in a way to create close contact between specific atoms. The specific dimers are discussed below in Section 3.2. All the structures, output files, and some additional scripts are available in an accompanying repository.^40^

The interaction energies of dimers, *E*_int_, were obtained as
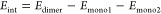
where *E*_dimer_, *E*_mono1_, and *E*_mono2_ are the energies of the dimer and the two monomers, respectively. Note that the structures of the monomers in the dimer and as isolated molecules are identical; for this reason, we use the term “interaction energy”. The error of the interaction energy with respect to the reference value *E*_*int*_^ref^ is then
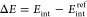


### 2.2 VASP Calculations

The majority of results for plane-wave PAW calculations were performed using the VASP.^6,34,36^ Version 5.4.1 was used for the calculations of the S22 and S66 data sets, and version 6.1.2 was used to calculate the binding curves of dimers with close contacts between specific atoms. We obtained results for the Perdew–Burke–Ernzerhof (PBE) exchange–correlation functional^19^ and for the Hartree–Fock (HF) method.^18^ The PBE-based Hard, Hard_GW, Standard, Standard_GW, and Soft potentials (available in the potpaw_PBE.52 VASP PAW data set version) were used. These are denoted by the suffices “_h”, “_h_GW”, none, “_GW”, and “_s”, respectively, in the PAW directory distributed with VASP, and their properties are summarized in Table ST5 in the Supporting Information, with more detailed information available in the output files.^40^ The general procedure used to construct the PAW data sets for VASP is discussed in ref (6). The “_GW” PAWs contain partial waves with energies of several hundred eV to improve the description of states with high energies; more details are given in ref (37).

Unless noted, we used PW basis-set cut-offs of 2000, 1600, and 1000 eV for the Hard (and h_GW), Standard (and _GW), and Soft PAW potentials, respectively. In order to minimize the interactions between periodic images, a large simulation cell with a side of 35 Å was used to gather the data for the S22 and S66 data sets and to perform distance scans. Residual spurious interactions between periodic images were further reduced using the dipole correction in VASP (IDIPOL = 4 tag in INCAR).^41^ The *k*-point sampling was performed only at the Γ-point. To avoid accidental partial occupancies of one-electron states, we used Gaussian smearing (ISMEAR = 0 in VASP) with a small smearing width of 0.01 eV (SIGMA tag in VASP). To increase the precision, we also set LASPH = .TRUE. in the VASP input to account for the aspherical contributions to the density gradient component of the exchange–correlation energy for the on-site PAW terms. The real-space Coulomb cutoff technique (HFRCUT tag in VASP) was not used for the HF calculations as we are using the same simulation cells for the dimer and monomers, and thus, the interaction energy is not affected by error due to Coulomb singularity.^42,43^

To help with the analysis of errors, we obtained the Bader^44^ and iterative Hirshfeld charges. The iterative Hirshfeld charges were calculated within VASP by an algorithm proposed by Bultinck et al.,^45,46^ as implemented by Bučko and co-workers (IVDW = 21 tag in INCAR(27,47)). The Bader charges were obtained using the Bader charge analysis code on the approximate all-electron density printed by VASP.^48−51^

### 2.3 Quantum Espresso Calculations

Further tests of PAW potentials were performed using the PWscf code of Quantum Espresso, version 7.2.^52−54^ Specifically, we tested the PAWs available in pslibrary.1.0.0,^10^ in both their “precision” and “efficiency” variants. For Quantum Espresso, the calculations were automated using the Atomic Simulation Environment, version 3.22.1.^55^ To reach a high precision, a setup similar to that used in VASP was used. In particular, the same structures with large simulation cells were employed. The PW basis-set cutoff was set to 147 Ry (around 2000 eV, ecutwfc = 147 settings in the input), and the cutoff for density was set to 588 Ry (tag ecutrho in the input). The convergence threshold on total energy for orbital optimization (tag conv_thr) was set to 1 × 10^–8^ a.u.

### 2.4 All Electron Calculations

The all-electron calculations were carried out using the Molpro^56,57^ and Turbomole^58^ packages. To reach a high precision of the results, we did not use the so-called resolution of identity approximation and set tight convergence criteria for the orbital and energy in Molpro (ORBITAL and ENERGY set to 1 × 10^–8^ a.u.) and for the wave function and density in Turbomole (1 × 10^–8^ a.u). Moreover, the precision of the integration grid was modified compared to the default options by setting the grid tag in Molpro to 1 × 10^–8^ and using the finest grid (value 7) in Turbomole. The interaction energies were obtained using Dunning’s aug-cc-pV*N*Z (*N* = D, T, Q, 5, and 6) basis sets; we denote them by the abbreviation AV*N*Z.^21,22,59,60^ When calculating the interaction energy for a specific dimer, all the calculations used the dimer basis set.^61^ The data obtained with the AV5Z basis set was used as a reference for the S22 and S66 data sets. We note that our PBE/AV5Z interaction energies for the S22 dimers are in a very good agreement with the results obtained using a large pc-4 basis set^62,63^ by Witte and co-workers;^29^ the RMSE is only 0.14 meV.

To help the analysis as well as to obtain precise categorization of the considered complexes, we used the symmetry-adapted perturbation theory (SAPT)^64^ results of Heßelmann for the S22 and S66 data sets.^65^ Specifically, the first-order electrostatic (*E*_elst_^(1)^) and second-order dispersion (*E*_disp_^(2)^) interaction energy components were obtained by localized asymptotically corrected PBE0 XC potential (LPBE0AC)^19,66^ and the exact-exchange KS response kernel (EXX).^67,68^

Finally, for analysis, atom–atom interaction energies were obtained using an interacting quantum atoms (IQA)^69,70^ scheme applied to PBE/AVTZ and HF/AVTZ densities. In the IQA scheme, the dimer interaction energy is written as a sum of atom–atom interaction energy contributions as follows
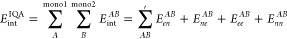
1where *A* and *B* represent atomic basins belonging to monomer 1 and monomer 2, respectively, as defined by the quantum theory of atoms in molecules (QTAIM).^44^ The *E*_int_^*AB*^ is decomposed into nuclear-electron (*E*_*ne*_^*AB*^ and *E*_*en*_^*AB*^), electron–electron (*E*_*ee*_^*AB*^), and nuclear–nuclear (*E*_*nn*_^*AB*^) contributions. The *E*_*ee*_^*AB*^ is further divided into a sum of Coulomb (*E*_*C*_^*AB*^) and XC (*E*_*XC*_^*AB*^) contributions. Therefore, the *E*_int_^IQA^ components can be categorized as
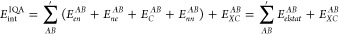
2where *E*_*elstat*_^*AB*^ and *E*_*XC*_^*AB*^ are the classical electrostatic and XC contributions to atom–atom interactions, respectively. All the PBE/AVTZ and HF/AVTZ densities for the IQA calculations were obtained by Molpro,^56,57^ and the IQA calculations were carried out using AIMAll.^71^

## 3 Results

### 3.1 S22 and S66 Databases

**Figure 1:**
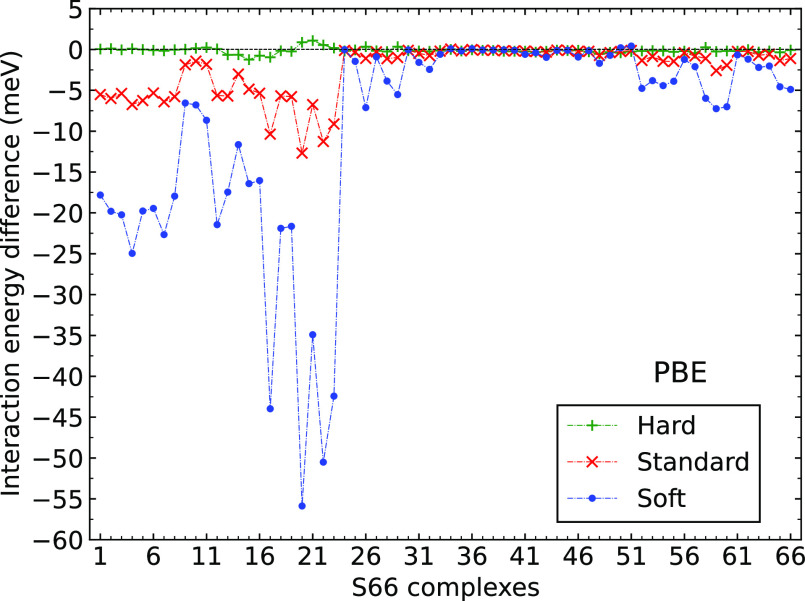
Errors of Hard, Standard, and Soft PAW potentials for the PBE interaction energies of S66 dimers.

We start with the errors of the different PAWs calculated for the PBE interaction energies of the S66 dimers, as shown in Figure 1 (the errors are also listed in Table ST8). As expected, the errors are the largest in the absolute value for the Soft PAWs and decrease when going to the Standard and Hard PAWs. The Hard PAWs produce results of essentially reference quality; the maximum deviation from the reference AV5Z result is ca. – 1.2 meV (see Table ST9). The Standard and Soft PAWs have negligible errors only for the complexes 35–51, which are dispersion-dominated. The largest errors of Standard and Soft potentials can be seen for the hydrogen-bonded complexes, i.e., complexes 1–23; the errors of the “mixed” complexes are smaller but still significant. The results of the _GW potentials are similar to the non-_GW variants, and we thus do not include them in Figure 1 (see Figures SF1 and SF2 for the variation of errors of Hard_GW and Standard_GW across the S66 data set, respectively). The results obtained for the S22 database (see Tables ST12 and ST13) are qualitatively similar, and we thus show them only in the Supporting Information (Figures SF3–SF6). Before analyzing the results in more detail, we comment on the errors observed for the HF method and for Gaussian basis sets.

The errors obtained for the HF method (Tables ST12 and ST13 for S66 and S22, respectively) are similar to those of PBE. The individual errors are shown in Figure SF7 in the Supporting Information, and in Table 1 we list the root-mean-square error (RMSE), mean absolute error (MAE), and mean error (MAE) on the whole S66 (see Tables ST11 and ST14 for the S22 data set). The small differences between HF and PBE can be illustrated by the overall RMSE for the Standard potential, that is, ca. 4.0 and 4.4 meV for PBE and HF, respectively. The largest difference in PBE and HF errors for Standard PAW occurs for the Uracil dimer (number 17), where PBE and HF give errors of −10.5 and −13 meV, respectively. For the Soft PAW, the largest difference between the PBE and HF errors is 8.7 meV for the acetic acid dimer (number 20), with the errors being −55.9 and −47.2 meV for PBE and HF, respectively. Despite the similarities, there are noticeable differences between the _GW and non-_GW PAWs. For example, the statistical errors are almost identical for PBE and HF when Hard_GW PAW is used (Table 1). In contrast, the errors somewhat increase for the Hard PAW when PBE is replaced with HF. Overall, the data show that the main cause of the error is likely identical for PBE and HF calculations. Moreover, the _GW PAWs are likely more reliable when using other functional than PBE.

**Table 1:** RMSE, MAE, and ME of Hard, Hard_GW, Standard, Standard_GW, and Soft Potentials as Well as AVDZ, AVTZ, and AVQZ Basis Sets with Respect to the AV5Z Basis Set on S66 Data for the PBE Functional and HF Method^a^

	PBE			HF		
potential/basis set	RMSE	MAE	ME	RMSE	MAE	ME
Hard	0.4	0.3	–0.1	0.9	0.7	–0.7
Hard_GW	0.5	0.4	–0.1	0.5	0.4	–0.2
Standard	4.0	2.5	–2.5	4.4	2.6	–2.6
Standard_GW	4.0	2.5	–2.5	4.3	2.7	–2.7
Soft	16.0	9.5	–9.5	14.8	8.7	–8.6
AVDZ	3.2	2.1	1.7	3.5	1.9	1.4
AVTZ	1.4	0.9	0.8	1.2	0.7	0.7
AVQZ	0.1	0.1	0.0	0.1	0.0	0.0

a The errors are in meV.

In Table 1, we also list the statistical errors obtained for the Gaussian basis sets from the AV*N*Z family; the individual values are tabulated in Tables ST8 and ST12 for PBE and HF methods, respectively. The data show that the errors of the AVQZ basis set are marginal; this is expected due to the exponential convergence of the total energy. The Hard and Hard_GW potentials give overall errors between those of the AVTZ and AVQZ basis sets. For the Hard potential, the errors are similar to those of the AVTZ basis set when HF energies are calculated. In terms of statistics, the Standard and Standard_GW lead to higher average errors compared with the counterpoise-corrected AVDZ values for the S66 database complexes. However, the situation is not so simple as the errors vary a lot between the different dimers, and in the following, we discuss how the errors depend on the character of the binding.

Let us now analyze the results in a more detail by considering statistical errors for different subsets of S66. The subsets that we use are hydrogen bonds (HB), π–π, π–σ, σ–σ, and “others”. With few exceptions, the HB and others correspond to the electrostatic and mixed groups of Řezáč et al.,^33^ respectively, and the groups involving σ and π bonding introduce a more fine-grained classification of the dispersion-dominated dimers. Our classification for the individual dimers, along with the original classification from ref (33), is given in Table ST8.

**Figure 2:**
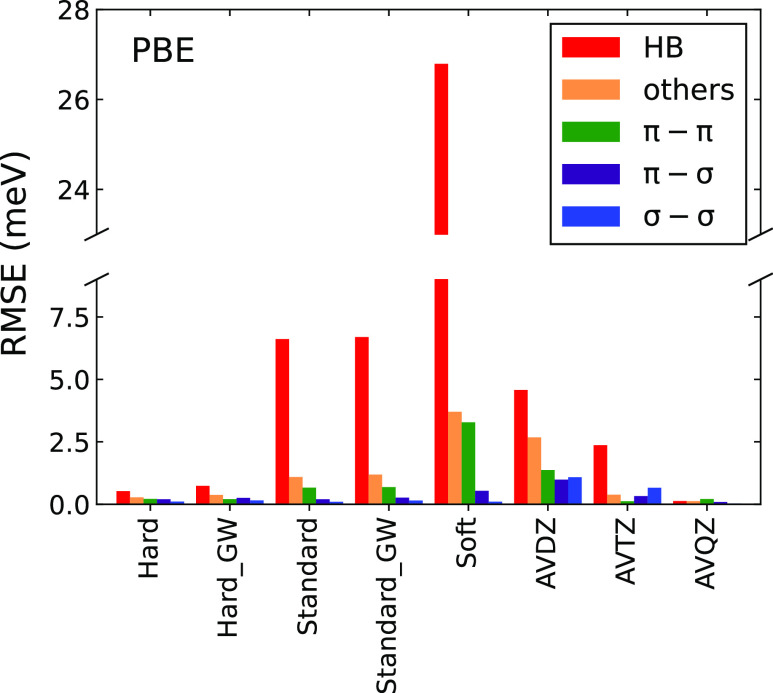
RMSE in meV of Hard, Hard_GW, Standard, Standard_GW, and Soft potentials, as well as AVDZ, AVTZ, and AVQZ basis sets with respect to AV5Z on the S66 data set for different subsets of the S66 data set.

The RMSEs of the different PAW potentials and the AV*N*Z basis sets for the different subsets of S66 are shown in Figure 2 and tabulated in Table ST15 of the Supporting Information. All the PAWs show a similar trend: the largest RMSE is observed for the HB complexes, followed by the others, the π–π, π–σ, and σ–σ groups. The errors for the HB subset are small and comparable to the errors of the other subsets only for the two Hard PAWs. For example, for the Hard PAW, the RMSE for the HB subset is around 0.5 meV, while the RMSEs for the π–π and π–σ subsets are close to 0.2 meV. The errors for the HB subset clearly dominate for the Standard and Soft PAWs; they are at least five-times larger than the RMSEs for the other subsets. Interestingly, the errors for the σ – σ subset are small for all of the PAWs, with RMSEs between 0.1 and 0.2 meV.

**Figure 3:**
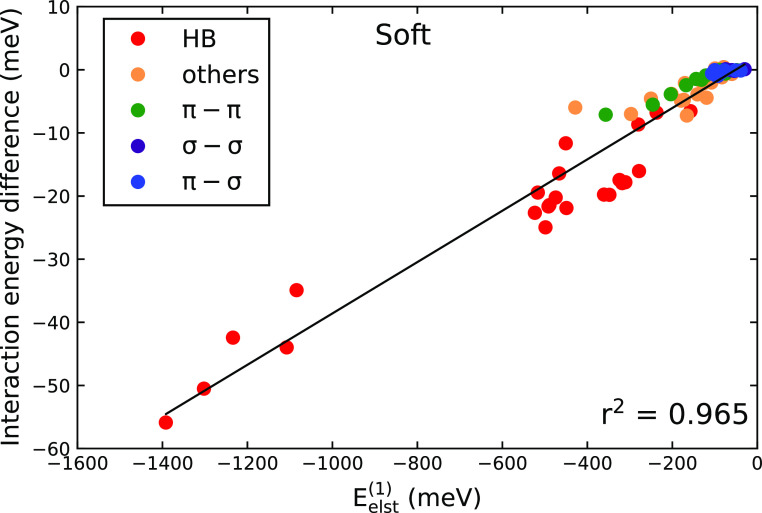
Dependence of the errors of Soft potential with respect to the PBE/AV5Z reference on the magnitude of the first-order electrostatic (*E*_elst_^(1)^) term obtained with the DFT-SAPT-EXX method for the S66 database.^66^ The points are colored according to the subset of S66 to which they belong.

The errors of the interaction energies for standard and Soft PAWs increase when going from the σ–σ to the HB subsets. This strongly suggests that the errors depend on the electrostatic contribution to the interaction energy. In order to quantitatively assess this relation, we compared the PAW errors to interaction energy components obtained by SAPT. This analysis shows a clear correlation between the PAW errors and the electrostatic component of the interaction energy, as illustrated in Figure 3 for the Soft PAW. We do not observe any significant correlations between the errors and other SAPT components.

The statistical quantities in Table 1 show that the errors of the Standard PAW are about 25% larger than those of the AVDZ basis set. However, comparing the RMSEs for the different subsets, one can see that only the RMSE of the HB subset is larger for Standard PAW than for the AVDZ basis set (Figure 2). The Standard PAW leads to a smaller RMSE for the rest of the subsets. In fact, for the σ–σ and σ–π subsets, the errors of the AVDZ basis set are even larger than those of the Soft PAW. Therefore, the errors of the AVDZ and other Gaussian basis sets are distributed more evenly than the PAW errors. As discussed by Witte et al.,^72^ the errors for the Gaussian basis sets tend to depend on the number of atoms in contact, while for the PAWs, we find the dominant role of the electrostatic component. Clearly, these two do not need to correlate with each other, leading to qualitative differences.

**Figure 4:**
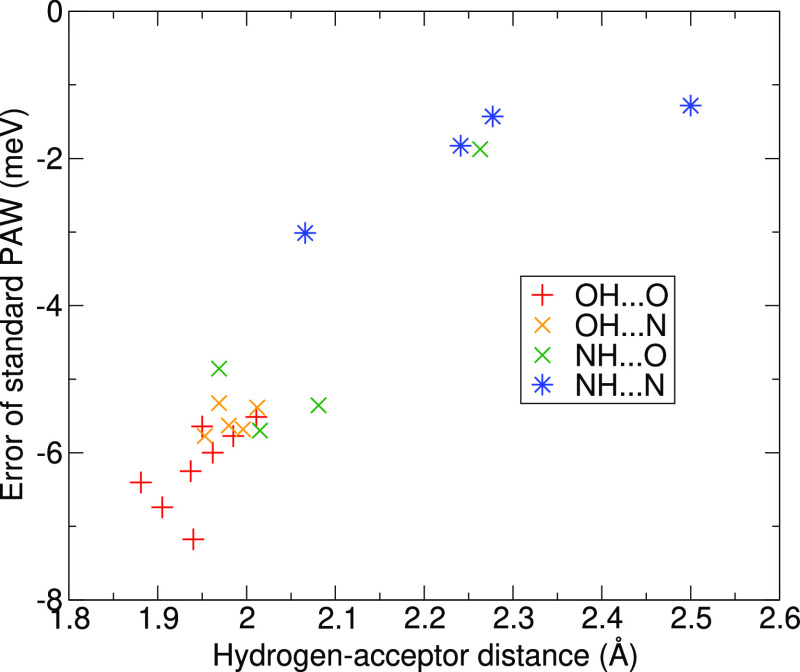
Errors of the interaction energies of the Soft PAWs for dimers with a single HB in the S22 and S66 data sets as a function of the distance between the donor hydrogen and the atom accepting the HB.

As the errors are the largest for the HB dimers, we now analyze them in more detail. First, we take dimers with a single HB from sets S22 and S66 and plot the errors for these dimers as a function of the distance between the hydrogen atom and the acceptor atom. The data are shown in Figure 4, divided into groups according to the accepting and donating atoms. One can see that the errors tend to increase in magnitude with the decrease in HB length. This is visible for all of the acceptor–donor pairs and also for each group individually. Moreover, the errors seem to be larger for the dimers involving oxygen as the donor and as the acceptor compared to the dimers containing nitrogen as both the donor and acceptor. However, extracting more detail is difficult as the HB lengths differ for the two groups, and we return to the distance dependence of the error later.

As a final step of the analysis of the S66 data, we considered the errors of bifurcate complexes: dimers with two HBs. These are uracil–uracil, acetic acid dimer (AcOH–AcOH), acetamide dimer (AcNH_2_–AcNH_2_), acetic acid-uracil (AcOH-uracil), and acetamide-uracil (AcNH_2_-uracil), which are complexes 17, 20, 21, 22, and 23 in the S66 database. As one can see in Figure 1, these dimers have the largest errors. Moreover, they also show the largest differences between the HF and PBE errors. Table 2 lists the errors obtained for the Hard, Standard, and Soft PAWs for the bifurcated HB complexes together with the atoms involved in the HBs. The errors of Soft and Standard potentials in absolute value for the bifurcate complex decrease in the order AcOH···AcOH > AcOH···uracil > uracil···uracil > AcNH2···uracil > AcNH2···AcNH2. As with the systems with a single HB, the errors are clearly larger when the HBs involve oxygen as the donor than when the donor is nitrogen.

**Table 2:** Error of Hard, Standard, and Soft Potentials (in meV) for the Bifurcate Hydrogen-Bonded Complexes of the S66 Database as Well as the Atoms Involved in the Hydrogen Bonds

no.	complexes	Hard	Standard	Soft	linkage 1	linkage 2
17	uracil–uracil	–0.95	–10.36	–43.98	C–O···H–N	N–H···O–C
20	AcOH–AcOH	0.88	–12.68	–55.87	C–O···H–O	O–H···O–C
21	AcNH_2_–AcNH_2_	1.08	–6.75	–34.91	C–O···H–N	N–H···O–C
22	AcOH–uracil	0.55	–11.26	–50.51	C–O···H–N	O–H···O–C
23	AcNH_2_–uracil	0.17	–9.12	–42.43	C–O···H–N	N–H···O–C

### 3.2 Dimer Binding Curves

The data obtained for the S22 and S66 sets show a clear relationship between the PAW error and the magnitude of the electrostatic component of the interaction and the distance between the dimers. Moreover, the errors are the largest for molecules containing oxygen and nitrogen. To gain deeper insights into the errors, we constructed sets of dimers formed by small molecules and performed distance scans of the interaction energy and its error. Each set contains molecular dimers oriented in a way to create close contact between two specific atoms, such as O···O, O···H, C···O, H···H, or N···H. Several molecules were used for each atomic pair to understand how the error changes when the atoms are in a different environment. Moreover, we performed an IQA analysis to corroborate the results. In the following, we only discuss the results for the O···H and O···O contacts.

We start with the results obtained for the dimers involving an O···H contact, i.e., a HB involving oxygen as an acceptor. In our set, there are five dimers: a water dimer and two dimers where water is an HB donor, and the other molecules are CO_2_ and CO, with oxygen as the HB acceptor. Finally, water acts as an HB acceptor in two dimers, with the HB-donating molecules being ammonia and methane. The structures of all the dimers are shown in the Supporting Information in Figure SF8.

**Figure 5:**
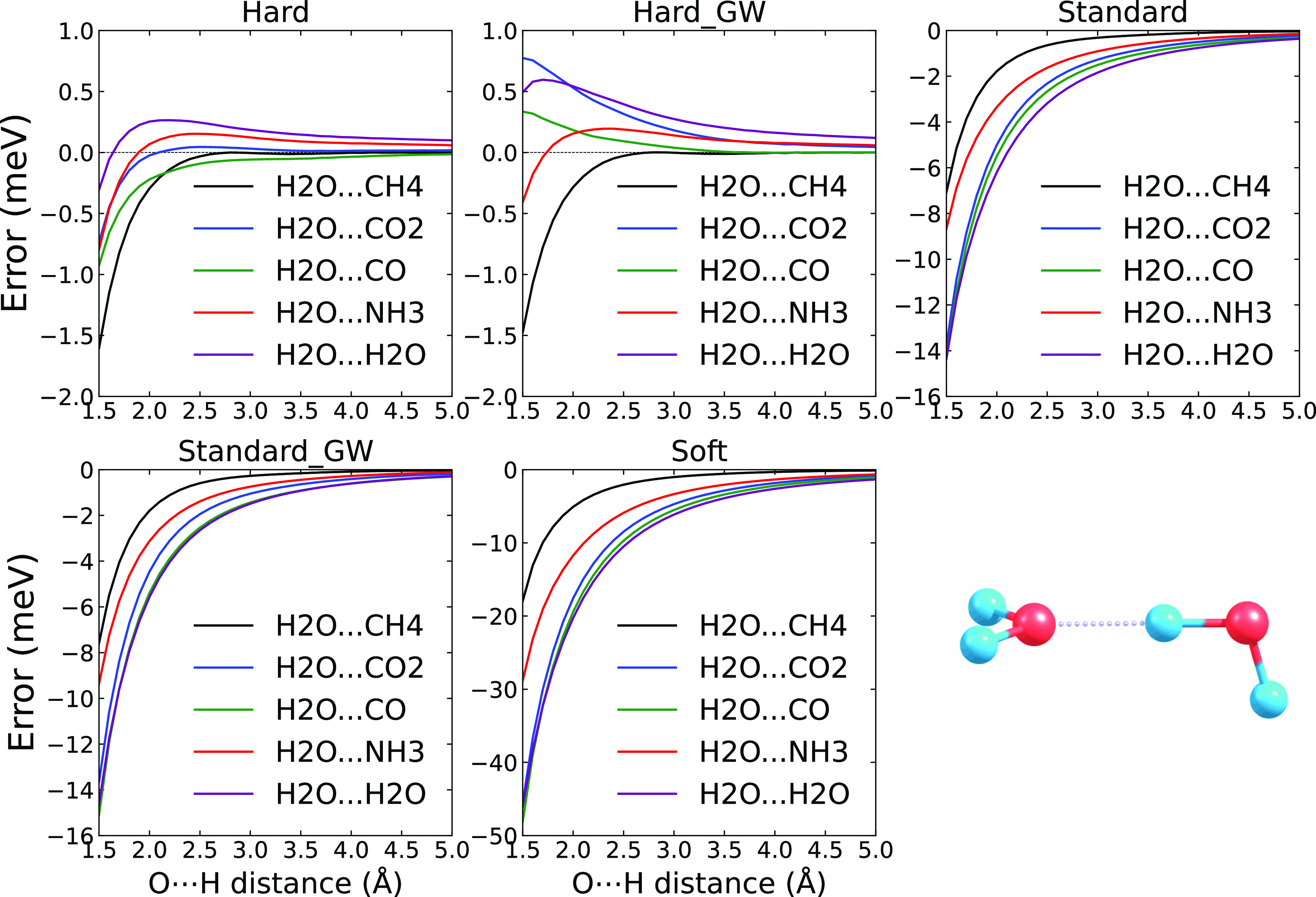
PBE potential energy curve errors of Hard, Hard_GW, Standard, Standard_GW, and Soft potentials (with respect to PBE/AV6Z) for the systems with an O···H contact in meV together with the structure of a water dimer.

Figure 5 shows the errors of the PBE interaction energies for the different PAW potentials with respect to the AV6Z basis set as a function of the distance between the bridging hydrogen and the HB acceptor. As expected, the errors are again the largest for the Soft PAW and reduce when going to the Hard and Hard_GW PAWs. The errors for the two Hard PAWs are less than 0.5 meV in absolute magnitude for most of the considered distances and only get larger for distances below ≈2 Å. For either of the Hard PAWs, the error seems to be a combination of a slowly decaying component, in most cases with a positive value, and a quickly decreasing negative contribution at short distances. In contrast, the errors of the Standard and Soft PAWs are negative for all the distances and decay monotonously with increasing donor–acceptor distance.

We now consider the errors for the three dimers where water accepts an HB. The errors clearly increase when going from methane over ammonia to water as the HB-donating molecule. This holds for the Standard PAWs and Soft PAWs for all of the distances and for the slowly decaying component of the Hard PAWs. In fact, the water–methane error apparently has only the short-range component, and the errors are close to zero above ∼3.0 Å.

**Figure 6:**
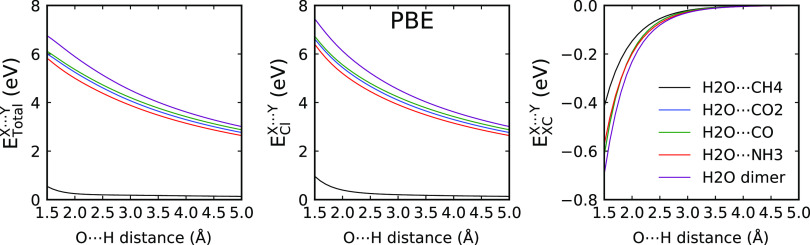
Total X···Y IQA interaction energy curve (*E*_total_^X···Y^) and its classical Coulomb (*E*_Cl_^X···Y^) and exchange–correlation (*E*_*XC*_^X···Y^) contributions for the dimers with an O···H contact. X and Y refer to the hydrogen atom acceptor and donor, respectively. All energies are in eV.

As the error is a combination of at least two contributions, it is useful to analyze the nature of the donor–acceptor interactions in the systems using the IQA approach. The IQA provides information about the classical electrostatic contribution to the interaction energy and exchange correlation contribution. The data are shown in Figure 6 together with the total interaction curve between the donor and acceptor atoms. One can see that for all of the systems except methane, the classical Coulomb interaction energy is large and has a slow decay. The exchange–correlation is much smaller and has a faster decay. The ordering of the dimers according to the error observed for Standard PAW is the same as the ordering according to the magnitude of the electrostatic interaction as provided by IQA. Therefore, the two contributions observed in the curve for the Hard PAW can be tentatively ascribed to originate from the Coulomb interaction (long-range part) and overlap-related terms (short-range).

We now turn to the dimers containing a direct O···O contact. The systems are CO_2_ dimer, O_2_ dimer, CO_2_···CO dimer, water dimer, and complexes of water with CO and CO_2_ molecules. The geometries are given in the Supporting Information, along with the figures of the structures in Figure SF9. Note that these structures do not necessarily correspond to the lowest energy ones, but such configurations can be important nevertheless. For example, a water dimer with two oxygens in direct contact appears in a high-pressure ice VIII phase with an interatomic distance of around 3.3 Å.

**Figure 7:**
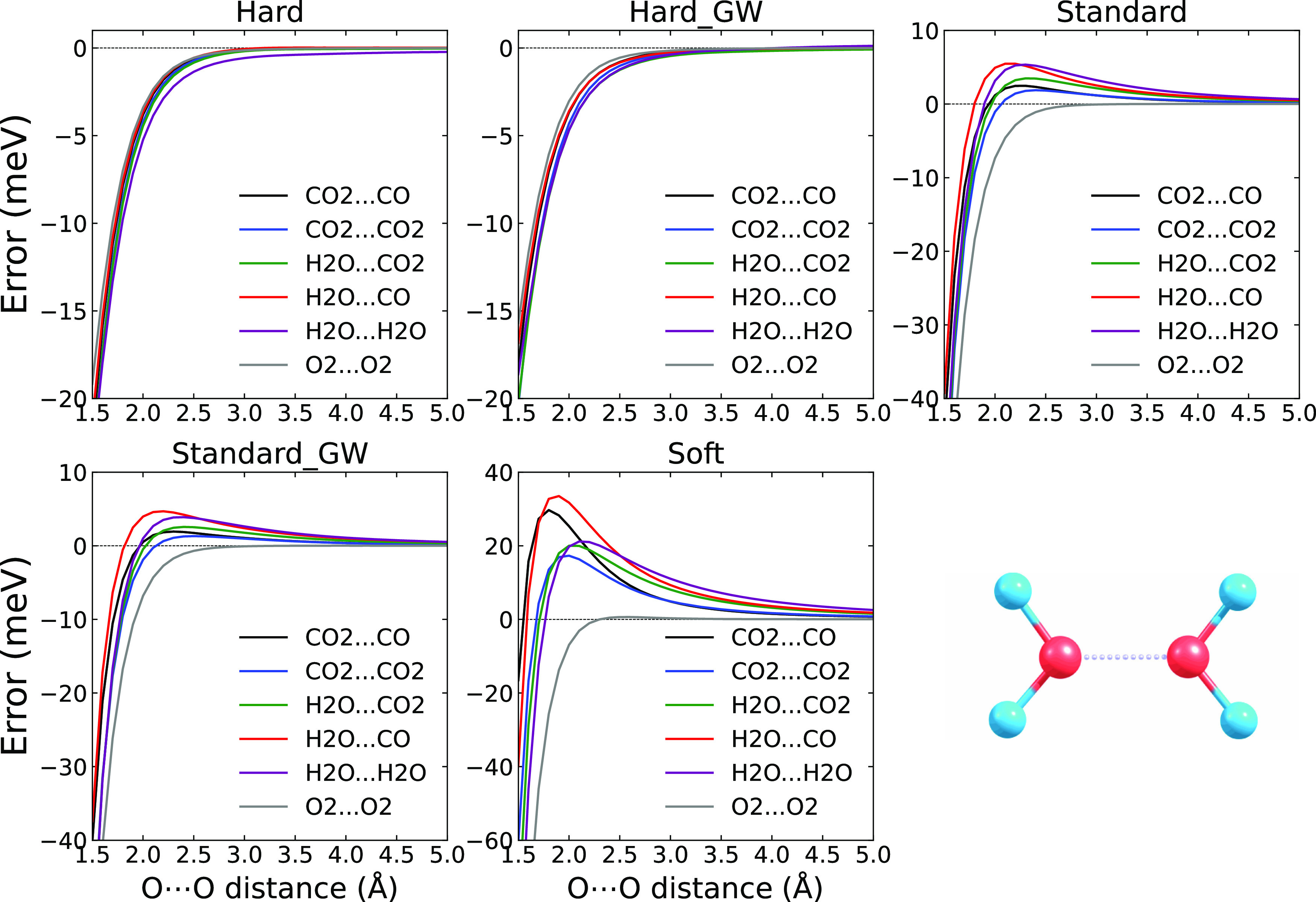
PBE potential energy curve errors of Hard, Hard_GW, Standard, Standard_GW, and Soft potentials (with respect to PBE/AV6Z) for the O···O contact dimers in meV. The bottom right panel shows the used structure of the water dimer with an O···O direct contact.

The errors of the Hard, Hard_GW, Standard, Standard_GW, and Soft PAW potentials obtained for the O···O contact systems are shown in Figure 7, together with an image of the water dimer structure as the representative of the dimers. As with the dimers with an O···H contact (or HB), two different sets of results appear. For the Hard PAWs, the error goes monotonously to zero with increasing distance between the molecules. For the Standard PAWs and Soft PAWs, the error is negative for short distances and positive for more distant configurations. Note that the O···H contact dimers showed the same characteristics, but the individual PAWs belonged to the opposite groups. Therefore, the overall error is most likely composed of the two components for all the PAWs. For the systems considered here, the short-range part is always negative, and the long-range one depends on the mutual orientation of the molecules so that it can be positive or negative, as discussed below.

**Figure 8:**
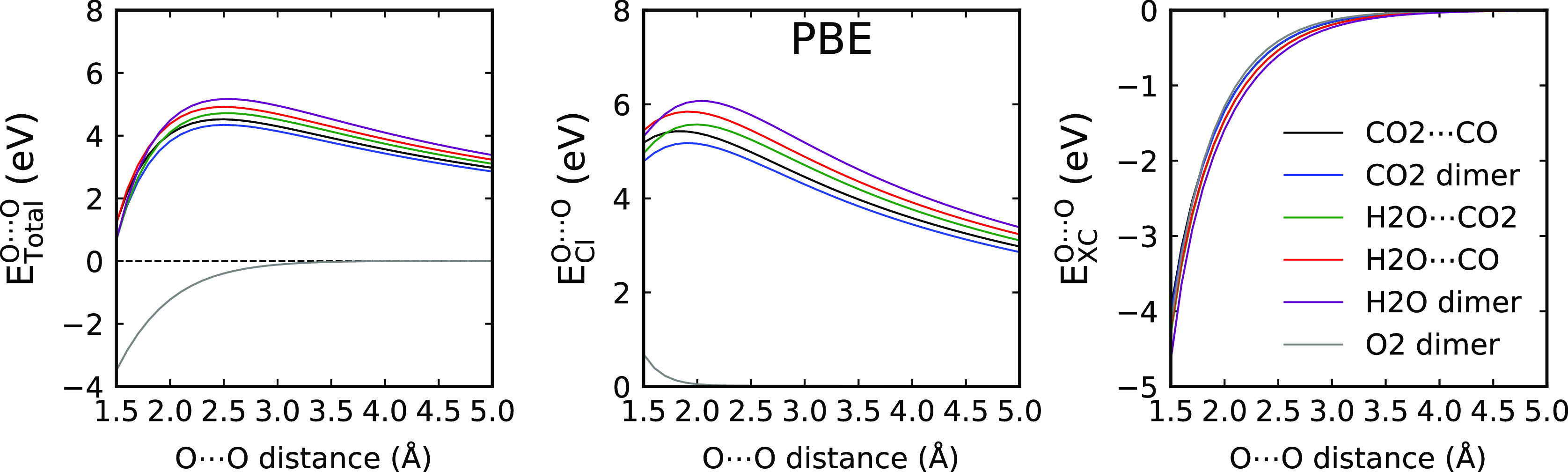
Total O···IQA interaction energy curve (*E*_total_^O···O^) and its classical Coulomb (*E*_Cl_^O···O^) and exchange–correlation (*E*_*XC*_^O···O^) contributions for the dimers with direct contact. All the energies are in eV.

We also observe considerable differences between the errors for the different molecules forming the dimers. The error has only the short-range component for the O_2_ dimer, while the long-range component is the largest for the water dimer. The ordering of the long-range errors agrees with the ordering of the oxygen–oxygen electrostatic interaction, as provided by the IQA analysis, as shown in Figure 8. Clearly, the long-range errors occur only for systems in which the oxygen has a nonzero partial charge. When the partial charge on oxygen is zero, like in the case of the O_2_ molecule, only the short-range (“overlap”) component occurs.

Overall, the binding curves show that the PAW errors have two components: a short-range one with a fast decay that is likely related to the density overlap of the molecules and a second, long-range one, which increases in magnitude with increasing electrostatic components of the interaction. Considering that the dimers are formed by neutral molecules, the leading-order electrostatic interaction is the dipole–dipole term. A dipole–dipole interaction could occur from errors in electron density caused by the use of the PAW approximation. This suggests that the error could be fitted by the function
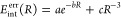
3in which the exponential function models the overlap component and the second term corresponds to the dipole–dipole interaction, *a*, *b*, and *c* are coefficients of the fit, and *R* refers to the intermolecular or interatomic distance.

**Figure 9:**
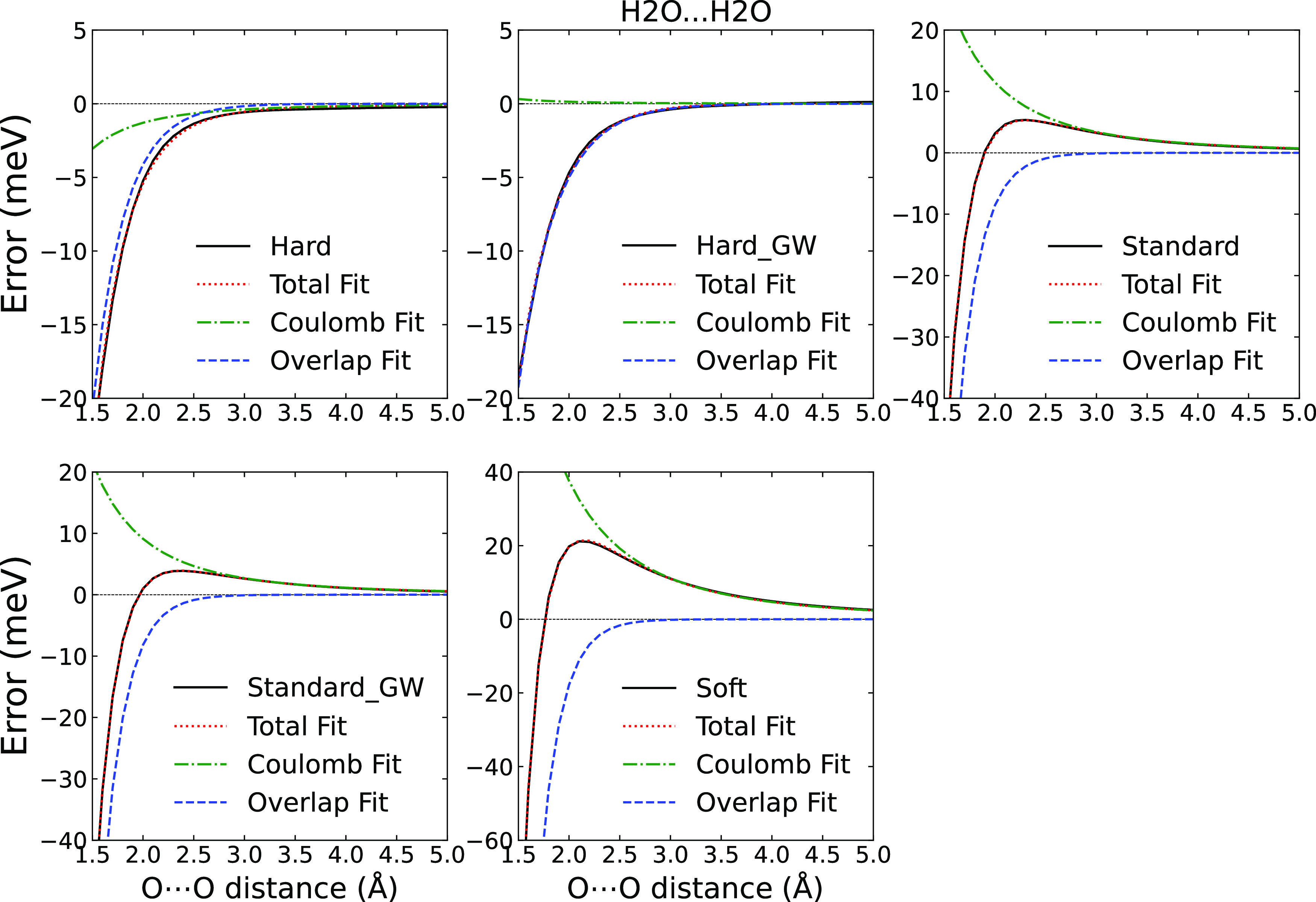
PBE potential energy curve errors of Hard, Hard_GW, Standard, Standard_GW, and Soft potentials (with respect to PBE/AV6Z) and their fitted curves to *a* exp (−*bR*) + *cR*^–3^ (total) as well as the Coulomb (*cR*^–3^) and overlap (*a* exp (−*bR*)) components of the errors for the O···O contact water dimer.

We show an example of the fit using eq 3 for the water dimer with O···O contact in Figure 9. The variable *R* is the distance between the oxygen atoms. The simple model fits the data very well for all of the PAW potentials. The Hard PAWs show almost negligible errors for the electrostatic component, and the exponential contribution dominates. The magnitude of the exponential component increases when going to Standard and Soft PAWs. For distances above ∼1.7 Å, the error due to the electrostatic component becomes larger than the overlap error. Moreover, the error clearly follows the *R*^–3^ asymptotic behavior and thus corresponds to the incorrect description of the dipole–dipole interaction stemming from incorrect electron density. This can be explicitly verified by calculating the dipole of the water molecule using the different PAW potentials. We indeed find that the dipole is 0.3747 eÅ for the Hard PAW but increases to 0.3801 eÅ for the Standard PAW and to 0.3914 eÅ for the Soft PAW. Finally, the fact that the long-range component is caused by the dipole–dipole term explains the changes in the errors when going from the hydrogen-bonded dimers (Figure 5) to the O···O contact (Figure 7). When the water molecule is flipped, the dipole turns as well, and the dipole–dipole interaction changes sign.

### 3.3 Performance of Other PAW Libraries

In principle, the errors observed for the VASP calculations could be affected by some choice made when implementing the PAW method or during the generation of PAW data sets. To verify that similar errors occur for other codes and PAW potentials, we calculated some of the results also using Quantum Espresso and the PAWs from pslibrary.1.0.0.^10,38^ Note that there are two available PAWs for oxygen in the pslibrary, and they differ from each other only in the radial cutoff used for the *l* = 1 projectors. The precision settings have a value of *r* = 1.35 au, while the efficiency has a cutoff of *r* = 1.45 au. These values are between those used for *p* states of oxygen in VASP; the values are 1.1 and 1.52 au for the Hard and Standard PAW, respectively.

**Figure 10:**
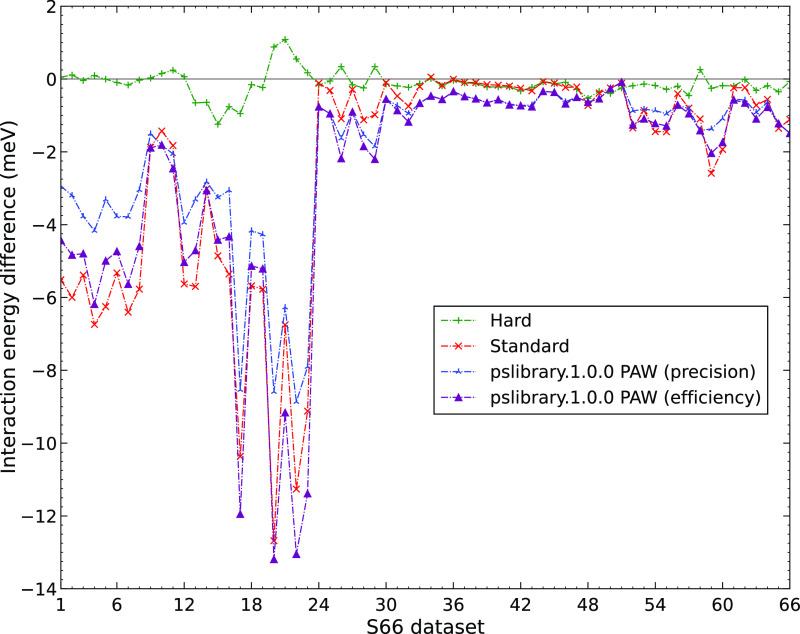
Errors of the PBE interaction energies for the S66 dimers as obtained by VASP using the Hard and Standard PAW potentials and Quantum Espresso using the “precision” and “efficiency” PAW potentials available in pslibrary.1.0.0.

The errors for the interaction energies of the S66 data set obtained for the pslibrary PAWs are shown in Figure 10. For comparison, we also included the data obtained with the Standard and Hard PAWs. One can see that the results obtained with the pslibrary potentials are qualitatively similar to those obtained with the Standard PAW potentials of VASP. Specifically, the errors are the largest for the hydrogen-bonded dimers and the smallest for the dimers bonded by dispersion. For the hydrogen-bonded systems, the errors for the precision PAWs are around 2/3 of the errors of the efficiency PAWs (average errors −4.2 vs −5.8 meV). Therefore, the errors correlate with the cutoff used for the *p* states, and PAWs with tighter settings would have to be used for the Quantum Espresso calculations to reduce the errors for the hydrogen-bonded dimers.^9^

**Figure 11:**
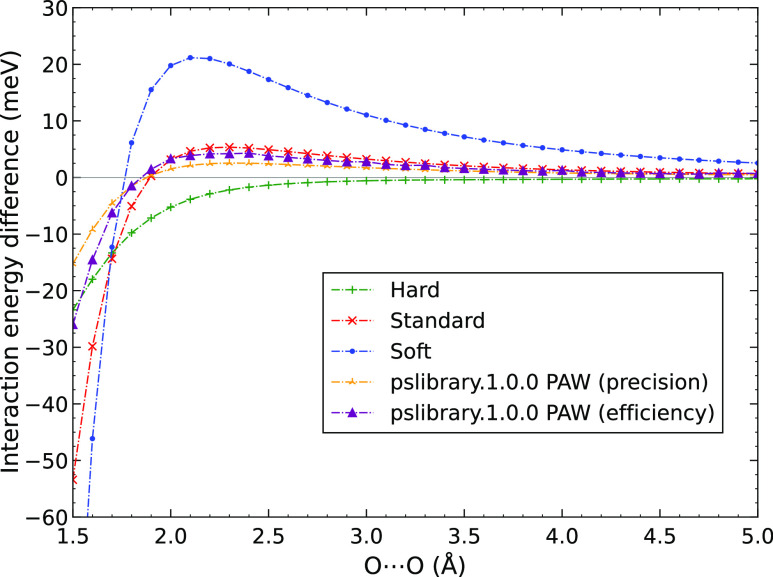
Errors for PBE interaction energy of the water–water dimer in the O–O contact configuration as obtained by VASP and Quantum Espresso using different PAW potentials.

We also calculated the interaction energy of the water dimer with oxygen–oxygen contact to test if the error for Quantum Espresso has a similar distance dependence to that observed for VASP. The errors of the interaction energies with respect to the AV6Z reference are shown in Figure 11 for both the VASP PAWs and the two pslibrary ones. As with the S66 data, we observe similar behavior between the two sets of PAWs, pointing to the same origin of the error. The precision PAWs clearly lead to smaller errors than the efficiency ones, both for the long-range component and the short-range part. Moreover, both of the pslibrary PAWs reach lower errors than the Standard PAW provided by VASP. The error of the precision PAWs is below that of the Hard PAWs for distances smaller than approximately 2.3 Å. However, above this distance, Hard PAWs give more precise results. As discussed, this is likely due to the smaller radial cut-offs used for the *p* states.

### 3.4 Electrostatic Correction

The long-range component of the PAW error comes from the Coulomb interaction, and the cause of the error, therefore, needs to be the incorrect density of the monomers. To assess the magnitude of the density errors, we considered several molecules from the S22 and S66 test sets and obtained charge density differences (Δρ) between Soft and Hard or Standard and Hard PAWs. For this comparison, we used the approximate all-electron densities printed by VASP by setting LAECHG =.TRUE. in INCAR.

**Figure 12:**
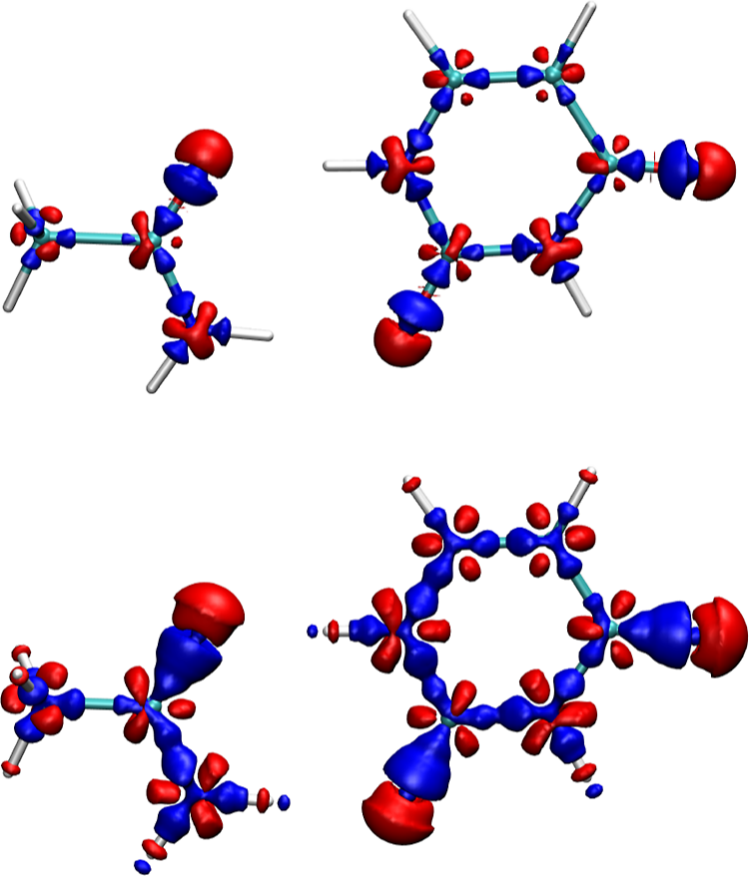
Electron density difference between Standard and Hard PAWs (top) and Soft and Hard PAWs (bottom) of the acetamide-uracil dimer. The red and blue isosurfaces are plotted at values of −0.02 and 0.02 Å^–3^.

Figure 12 shows an example of Δρ for the acetamide-uracil dimer for the Standard PAW (top) and Soft PAW (bottom) with the isosurfaces showing values of ±0.02 Å^–3^. There is a substantial electron density difference around the oxygen atom; the differences are smaller for carbon and nitrogen, while the density differences around hydrogens are visible only for the Soft PAW. The density difference for oxygen resembles a *p*-like function and thus has a dipole moment (see Figure SF10 as well). This is consistent with our previous findings that the interaction energy error can be fitted with a dipole–dipole term and the observation that the error correlates with the distance between donor–acceptor atoms. We find similar electron density errors around oxygen atoms in other molecules, and the density errors are of comparable size for nitrogen atoms with two neighbors or with three neighbors in a structure similar to ammonia (i.e., not all four atoms in the plane). When the nitrogen atom lies in the plane containing its three neighbors, as in the uracil shown in Figure 12, the overall density error lacks an out-of-plane dipole. The density error in the planar structure resembles the sum of three dipoles, each pointing in the direction of a covalent bond to one of the neighbors. The density differences for carbon atoms also resemble a sum of dipole like density errors contributed by each chemical bond. Therefore, the density errors have no or very small dipoles around carbon atoms for the molecules in the S22 and S66 data sets. Overall, these observations are consistent with the interaction energy errors calculated for the S22 and S66 dimers.

We now consider the part of the interaction energy due to density–density electrostatic interactions and use it to derive several formulas that can be used to quantify and correct the observed errors. First, the electrostatic interaction between the electron densities of two monomers (ρ_A_ and ρ_B_) is
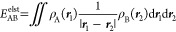
4

We define a density error of monomer A as
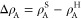
5where ρ_A_^S^ and ρ_A_^H^ are, respectively, the densities obtained by a more approximate PAW potential “S” (such as Standard or Soft) and a more precise one, such as that calculated using the Hard PAW potential. The density error for monomer B is defined analogously.

The error in the density–density electrostatic component of the interaction energy is
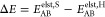
6the additional index compared to eq 4 indicates that the first energy is calculated with the approximate density and the second with the precise one. Using the relations for the electrostatic interaction of two electron densities (eq 4) and for the density errors of monomers A and B (eq 5) in eq 6, we obtain
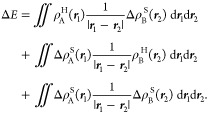
7This can be further simplified if we use an expression for the electrostatic potential created by electron density  and define *V*_B_^H^(**r**_1_) in a similar way. We obtain
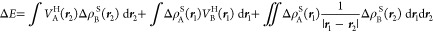
8The first two terms give the interaction of the density error of one monomer with the potential of the other monomer; the last term then describes the interaction of the two density errors. The last term is likely to be smaller compared with the first two contributions. If we neglect it, we obtain
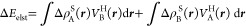
9which is the first relation that we use to compare to the actual error of different PAWs. This equation can be readily tested for VASP as all of the required quantities (electron densities and Hartree potential) can be written by the code and processed by an external program.

The form of the error suggests several ways to estimate it using more approximate means. One possibility is to approximate the electrostatic potential of a molecule by using atom-centered partial charges as
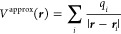
10where *r*_*i*_ are the positions of the atoms and *q*_*i*_ are the partial charges. Moreover, the density error can be approximately expanded to atom-centered multipole moments. In this work, we use only the dipole moment. Using these two approximations in eq 8 gives an approximate formula for the interaction energy error
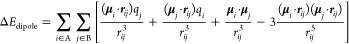
11where the indices *i* and *j* run over atoms in monomers A and B, respectively, μ_*i*_ are the dipoles assigned to atom *i*, and ***r***_*ij*_ is the vector between atoms *i* and *j*. Note that in practice, we use point charges obtained for the approximate PAW potentials and not for the more precise PAWs in eq 11 as we want to avoid calculations with the precise PAWs.

There are several methods that can be used to obtain atomic charges. We tested three of them here: Hirshfeld, iterative Hirshfeld, and Bader schemes. The main reason for choosing these approaches is that they are accessible from the VASP. Initial tests of the Hirshfeld scheme showed unsatisfactory charges for water (too small). Therefore, we discuss only the results obtained with the iterative version, for which we obtained more satisfactory results.

From the elements that are present in the S22 and S66 sets (hydrogen, carbon, nitrogen, and oxygen), the density errors are the largest for oxygen and nitrogen, and we, therefore, only set the dipoles of the density error for these atoms in eq 11. To obtain the magnitudes of the dipole moments of the density errors for oxygen and nitrogen, we calculated the dipole moments of water, formaldehyde, and methylamine using the different PAW potentials. Water was used to obtain dipole errors for oxygen with two neighbors and formaldehyde for oxygen with a single neighbor. The magnitude obtained with the Hard PAW was taken as a reference, and the difference between Standard and Soft PAWs was the sought dipole error magnitude. These values are summarized in Table 3.

**Table 3:** Atom-Centered Dipole Moment Errors Used in the Correction Scheme

element	no. neighbors	PAW	dipole error (eÅ)
O	1	Standard	0.0102
O	2	Standard	0.0076
O	1	Soft	0.0385
O	2	Soft	0.0240
N		Standard	0.0041
N		Soft	0.0161

The density errors resemble a dipole directed along an axis of rotation of the molecule (if there is exactly one). To obtain the direction of the dipole error vector, we first calculate the sum of normalized vectors for neighboring atoms *j*
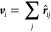
12We then check the length of ***v***_*i*_ as a small value indicates that atom *i* and its neighbors lie approximately in the same plane and the dipole moment of the density error, at least for the molecules considered here, is small. As mentioned above, this can occur for nitrogen, e.g., in uracil. If the length of ***v*** is equal to or above 0.75, we normalize it and use it as the dipole direction. The final dipole error is then obtained by multiplying the direction with the appropriate magnitude.

**Figure 13:**
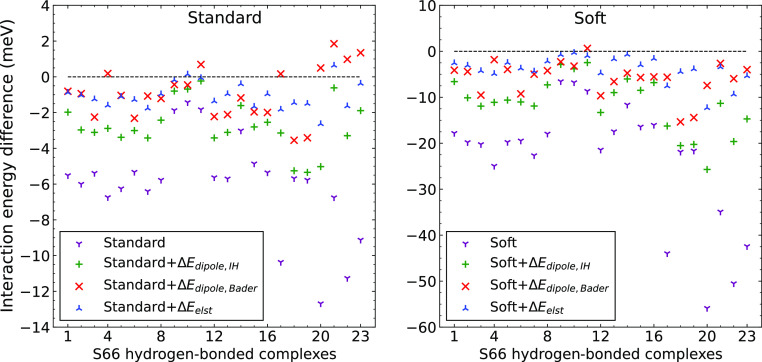
Errors of PBE interaction energies obtained for Standard and Soft PAWs for the HB-bonded complexes of the S66 data set. Bare data are shown with dots and corrected for the electrostatic error using various schemes: Δ*E*_dipole,Bader_ and Δ*E*_dipole,IH_ were calculated using eq 11 with Bader and iterative Hirshfeld charges, respectively, while Δ*E*_elst_ corrections were obtained using eq 9.

We used the models defined in eqs 9 and 11 to correct the errors of Standard and Soft PAW potentials on the S66 data set. The bare and corrected errors of the Standard and Soft PAW potentials are shown in Figure 13 for hydrogen-bonded dimers, and the average errors on the whole S66 set and its subsets are given in Table 4. All the corrections significantly reduce the errors of the two PAW potentials. The lowest average errors are observed for the least approximate density-based correction; the average errors for hydrogen-bonded dimers are reduced by a factor of ≈6 both for the Standard and Soft PAWs. The Bader-charges-based correction leads to similar average errors as the density correction for the Standard PAW but gives larger average errors for the Soft PAWs. One possible reason is that the density errors are larger for the Soft PAWs, and they are less well approximated using only the dipole term employed in the Bader-based correction. Therefore, terms beyond dipole for the charge density error and beyond monopole for the charge density are likely needed to improve the results. In any case, the Bader-corrected Soft PAW leads to similar errors as the uncorrected Standard PAW; at least for the hydrogen-bonded systems, the reduction of errors is less significant for the mixed and dispersion-bonded subsets.

**Table 4:** Mean Bare and Corrected Errors (in meV) with Respect to AV5Z for the Soft and Standard PAW Potentials for the S66 Test Set and Its Subsets^e^

PAW	correction	S66	HB	M	D
Standard	none^a^	–2.5	–6.0	–0.9	–0.3
	Δ*E*_elst_^b^	–0.6	–1.0	–0.4	–0.3
	Δ*E*_dipole,Bader_^c^	–0.6	–0.9	–0.6	–0.3
	Δ*E*_dipole,IH_^d^	–1.1	–2.7	–0.6	0.0
Soft	none	–9.4	–23.4	–2.9	–1.2
	Δ*E*_elst_	–1.7	–3.8	–0.8	–0.5
	Δ*E*_dipole, Bader_	–3.1	–5.9	–2.1	–1.2
	Δ*E*_dipole,IH_	–4.5	–11.4	–1.7	–0.1

a The bare PAW potential error.

b Using correction of eq 9.

c Using eq 11 and Bader atomic charges.

d Using eq 11 and iterative Hirshfeld atomic charges.

e HB, M, and D refer to hydrogen-bonded, mixed electrostatic-dispersion, and dispersion stabilized complexes.

The correction based on the iterative Hirshfeld charges is overall less efficient compared to the Bader-based scheme. Specifically, for the hydrogen-bonded dimers, the iterative Hirshfeld corrections are always smaller than those that use the Bader charges (Figure 13). The correction with iterative Hirshfeld charges is particularly small for dimers 18 and 19, which involve pyridine interacting with water and methanol, respectively; see Figure 13. Also, in this case, the iterative Hirshfeld charges are lower than the Bader ones. The scheme utilizing iterative Hirshfeld charges reduces the errors for the mixed and dispersion-bonded groups in a similar way to the Bader-based method.

**Figure 14:**
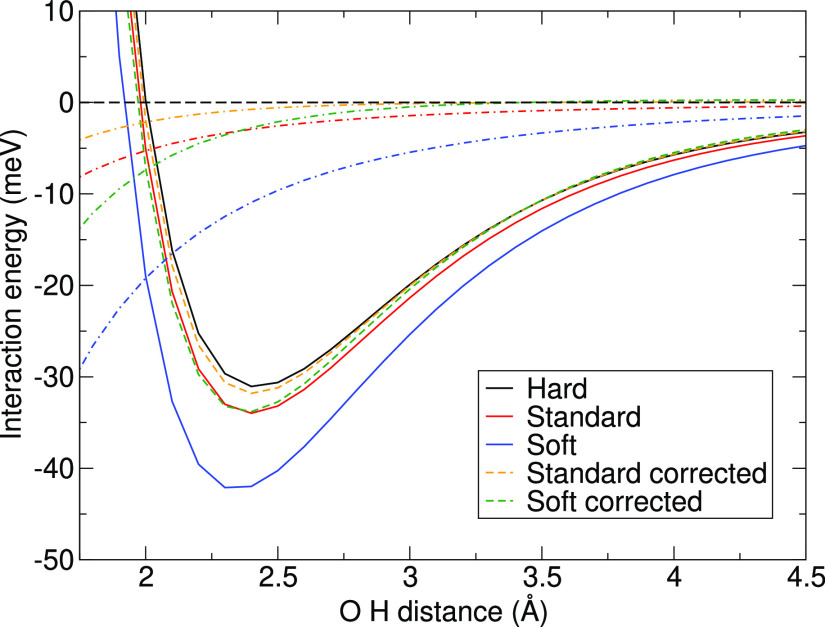
Binding curves of water and CO dimer were obtained for the Hard, Standard, and Soft PAW data sets (solid lines), and the data with correction according to eq 11 was applied to the Standard and Soft data (dashed lines). The dashed–dotted lines show the difference from the result obtained for the Hard PAW, which is taken as a reference. The iterative Hirshfeld charges calculated by VASP were used to calculate the correction.

To illustrate how the correction scheme performs for binding curves, we show data for the water-CO dimer with O···H contact in Figure 14. For the Standard and Soft PAWs, we give the curves without as well as with the correction according to eq 11, employing iterative Hirshfeld charges and oxygen-centered dipoles tabulated in Table 3. Moreover, we show the errors of the different settings with respect to the Hard PAWs by the dashed–dotted curves. One can see that the corrections considerably reduce the errors of both Soft and Standard PAWs for all of the distances. Around the equilibrium distance (around 2.5 Å), the error of the Soft PAW is reduced from around −9.6 to −2.1 meV. Moreover, the corrected Soft PAW has clearly better asymptotic behavior compared to the uncorrected one; the correction brings it to within 0.5 meV of the Hard PAW reference for distances above 3.0 Å. The errors of the corrected Soft PAW are even smaller than the errors of the uncorrected Standard PAWs for distances larger than the equilibrium distance. Also note that the interaction energy minimum on the Soft PAW curve is shifted to smaller distances by around 0.1 Å, and the correction partly remedies this issue. For the Standard PAW, the error at equilibrium decreases from −2.6 to −0.6 meV, and the difference to the Hard data for distances above 3 Å is around 0.1 meV or smaller.

## 4 Summary and Conclusions

We used the interaction energies of molecular dimers to test the precision of several PAW data sets, focusing primarily on those supplied with the VASP code. In general, we obtain similar qualitative results as obtained by previous tests,^14,25,26^ that is, we find that errors are larger for oxygen and nitrogen compared to carbon and hydrogen. However, compared to the previous works, we clearly see that the errors originate from incorrect electron density, leading to differences in electrostatic interactions.

We identify two components of the error: a short-range one with an exponential decay and a long-range one with an algebraic decay. The first one is less important for typical bond lengths of weakly bonded dimers, but, of course, it will affect atomization energies, covalent bond lengths, or very short HBs. The long-range error originates from errors in electron density that appear for more approximate PAW potentials (Standard, Soft, ...). For the molecules studied here, the density errors can be described as a sum of *p*-like functions centered at a given atom, with each function associated with one covalent bond. The total density error can be mostly *p*-like, such as for water or ammonia, or have a higher electrostatic moment, such as for nitrogen atoms in uracil or carbon atoms. This shape of the density error suggests a way to correct the error in the interaction energy. The density error can be modeled by atom-centered dipoles that interact electrostatically with the rest of the system. Moreover, the rest of the system can be approximated by atom-centered point charges. Even such simple corrections reduce the errors to around one-half for most hydrogen-bonded dimers. They are less efficient for the dispersion-bonded dimers where the density errors resemble a higher multipole, but an extension to these cases can be done by, e.g., assigning a density error to each covalent bond.

The proposed corrections for the long-range component are simple to implement and can be used to process an output of existing calculations or to correct the energies (and forces) on the fly. The only nontrivial requirement is the point charges, even though the charge density of the system could be used directly as well. Currently, we obtain Bader charges from the all-electron density produced by the VASP using an external tool. This is less convenient compared to the calculation of the iterative Hirshfeld charges, which can be done directly in the VASP code.^73^ However, the iterative Hirshfeld charges lead to worse results compared to the Bader charges, at least for the hydrogen-bonded systems. The short-range component, extending to some 1.0–1.5 Å from nuclei, could be modeled using exponential potentials such as those used in force fields or by a general machine learned force field. The latter approach could be also used to train and then predict the density errors in a similar spirit to learning total density.^74^

We demonstrated reduction of the error for molecular dimers, but the scheme can be easily extended to molecular solids or molecular adsorption, for which Standard PAWs are widely employed. Moreover, we expect that the origin of the error is likely identical for pseudopotentials, and the correction could be used for them as well. Finally, as the simple correction reduces the errors of Soft PAWs to a level of Standard PAWs, it suggests that the corrected Soft PAWs could be used for situations in which Standard PAWs are currently sufficiently precise. Similarly, the corrected Standard PAWs almost reach the precision of the Hard PAWs. Therefore, a general correction could allow one to reduce the computational cost of calculations or increase the accessible system size without a significant loss of precision. For dimers, we observe that the computational cost of the calculations depends on the basis-set cutoff *E*_cut_ as *E*_cut_^*n*^ with *n* between 1.0 and 1.5. Therefore, using PAWs with a cutoff of around 300 eV instead of harder PAWs with a cutoff of around 900 eV would reduce the computational cost by around a factor of 3. This reduction is useful for applications such as crystal structure prediction or the molecular dynamics of complex systems.^75,76^

Overall, we showed that the interaction energies of dimers are a suitable tool to assess the quality of PAW potentials and similar methods. Moreover, the errors originate from electron density, which can be used to visualize the deviations from reference. Finally, we tested a simple model for the error that could be used to validate the setup of users or partially correct for the error.

## Supporting Information Available

File with interaction energies and their errors for the S22 and S66 complexes as well as additional graphs, tables, and figures (PDF)
