# Supplementary material for: Using Noncovalent Interactions to Test the Precision of Projector-Augmented Wave Data Sets
Source: J Chem Theory Comput. 2023 Dec 1;19(23):8871–85. doi: 10.1021/acs.jctc.3c00930 (PMC10720388; doi:10.1021/acs.jctc.3c00930)
Supplement: Supplementary file 1 — ct3c00930_si_001.pdf [file ct3c00930_si_001.pdf]

Supporting Information:
Using Non-covalent Interactions to Test Precision of Projector-Augmented Wave Data Sets

Sirous Yourdkhani[1,*] and Jiří Klimeš[1,†]

[1] *Department of Chemical Physics and Optics, Faculty of Mathematics and Physics, Charles University, CZ-12116 Prague 2, Czech Republic*

[*] Electronic address: yourdkhani.sirous@karlov.mff.cuni.cz

[†] Electronic address: Jiri.Klimes@mff.cuni.cz

List of Figures

SF1 Variations in errors of Hard_GW potential across S66 dataset. 7

SF2 Variations in error of Standard_GW potential across S66 dataset. 7

SF3 Variations of errors of Hard and Hard_GW potentials across S22 dataset. 8

SF4 Variations of errors of Standard and Standard_GW potentials across S22 dataset. 8

SF5 Variations of error of Soft potential across S22 dataset. 9

SF6 Variations in error of Hard, Standard and Soft potentials across S22 dataset for PBE functional. 10

SF7 Variations in error of Hard, Standard and Soft potentials across S66 dataset for PBE method. 11

SF8 Hydrogen-bonded sample complexes. 11

SF9 The O$\cdots$O direct contact complexes. 12

SF10 Electron density difference between Soft and Hard PAWs of water molecule. The red and blue iso-surfaces are plotted at values of -0.02 and 0.02 Å^{-3}. 13

List of Tables

ST1 The parameters of Hard potentials (`POTCAR`) used in the present study. Valence denotes which orbitals are considered as valence. r_c^l and r_{comp}^l refers to the cut-off radii of partial waves and radii of pseudo-partial-waves, respectively. Ecut† refers to the energy cut-off of the PAW potential. In all the cases two partial waves are used for the s and p orbitals. 14

ST2 The parameters of Hard_GW potentials (`POTCAR`) used in the present study. Valence denotes which orbitals are considered as valence. r_c^l and r_{comp}^l refers to the cut-off radii of partial waves and radii of pseudo-partial-waves, respectively. Ecut† refers to the energy cut-off of the PAW potential. In all the cases, but hydrogen, three partial waves are used for the s and p orbitals. For 1s of hydrogen atom only two partial waves are used. 14

ST3 The parameters of Standard potentials (POTCAR) used in the present study. Valence denotes which orbitals are considered as valence. r_c^l and r_{comp}^l refers to the cut-off radii of partial waves and radii of pseudo-partial-waves, respectively. Ecut† refers to the energy cut-off of the PAW potential. In all the cases two partial waves are used for the s and p orbitals. 15

ST4 The parameters of Standard_*GW* potentials (POTCAR) used in the present study. Valence denotes which orbitals are considered as valence. r_c^l and r_{comp}^l refers to the cut-off radii of partial waves and radii of pseudo-partial-waves, respectively. Ecut† refers to the energy cut-off of the PAW potential. In all the cases two partial waves are used for the s and p orbitals. 15

ST5 The parameters of Soft potentials (POTCAR) used in the present study. Valence denotes which orbitals are considered as valence. r_c^l and r_{comp}^l refers to the cut-off radii of partial waves and radii of pseudo-partial-waves, respectively. Ecut† refers to the energy cut-off of the PAW potential. In all the cases two partial waves are used for the s and p orbitals. 16

ST6 The parameters of pslibrary.1.0.0 PAW (precision) potentials used in the present study. Valence denotes which orbitals are considered as valence. r_{loc}, r_l, r_{core}, and r_{sph} refer to the radius of the local potential, the cut-off radii for the *s*, *p*, *d* orbitals, the pseudization radius of the core charge, and the values of radius of the PAW sphere respectively. Ecut† refers to the energy cut-off of the PAW potential (in eV). 16

ST7 The parameters of pslibrary.1.0.0 PAW (efficiency) potentials used in the present study. Valence denotes which orbitals are considered as valence. r_{loc}, r_l, r_{core}, and r_{sph} refer to the radius of the local potential, the cut-off radii for the *s*, *p*, *d* orbitals, the pseudization radius of the core charge, and the values of radius of the PAW sphere respectively. Ecut† refers to the energy cut-off of the PAW potential (in eV). 17

ST8 Errors of Hard, Hard_GW, Standard, Standard_GW and Soft potentials as well as AVDZ, AVTZ, and AVQZ with respect to AV5Z method on S66 data for PBE functional. The errors are in meV. 18

ST9 The maximum absolute error of Hard, Hard_GW, Standard, Standard_GW and Soft as well as AVDZ, AVTZ and AVQZ for HF method and PBE functionl on S66 database. The errors are in meV. 20

ST10 Errors of Hard, Hard_GW, Standard, Standard_GW and Soft potentials as well as AVDZ, AVTZ, and AVQZ with respect to AV5Z method on S22 data for PBE functional. The errors are in meV. 20

ST11 Root mean square error (RMSE), mean absolute error (MAE) and average error (AVG) of Hard, Hard_GW, Standard, Standard_GW and Soft potentials as well as AVDZ, AVTZ, and AVQZ basis sets with respect to AV5Z basis set on S22 data for PBE functional. The errors are in meV. 22

ST12 Errors of Hard, Hard_GW, Standard, Standard_GW and Soft potentials as well as AVDZ, AVTZ, and AVQZ with respect to AV5Z method on S66 data for HF method. The errors are in meV. 23

ST13 Errors of Hard, Hard_GW, Standard, Standard_GW and Soft potentials as well as AVDZ, AVTZ, and AVQZ with respect to AV5Z method on S22 data for HF method. The errors are in meV. 25

ST14 Root mean square error (RMSE), mean absolute error (MAE) and average error (AVG) of Hard, Hard_GW, Standard, Standard_GW and Soft potentials as well as AVDZ, AVTZ, and AVQZ basis sets with respect to AV5Z basis set on S22 data for HF method. The errors are in meV. 26

ST15 Root mean square error (RMSE), mean absolute error (MAE) and average error (AVG) of Hard, Hard_GW, Standard, Standard_GW and Soft potentials as well as AVDZ, AVTZ, and AVQZ basis sets with respect to AV5Z basis set on S66 data for PBE functional for five types of complexes based on the geometrical orientations of monomers: hydrogen bonded (HB), aromatic-aromatic ($\pi - \pi$), aromatic-aliphatic ($\pi - \sigma$), aliphatic-aliphatic ($\sigma - \sigma$) and others. The errors are in meV. 27

ST16 Root mean square error (RMSE), mean absolute error (MAE) and average error (AVG) of Hard, Hard_GW, Standard, Standard_GW and Soft potentials as well as AVDZ, AVTZ, and AVQZ basis sets with respect to AV5Z basis set on S66 data for HF functional for four types of complexes based on the geometrical orientations of monomers: hydrogen bonded (HB), aromatic-aromatic ($\pi - \pi$), aromatic-aliphatic ($\pi - \sigma$), aliphatic-aliphatic ($\sigma - \sigma$) and others. The errors are in meV. 28

ST17 The maximum absolute error of Hard, Hard_GW, Standard, Standard_GW and Soft as well as AVDZ, AVTZ and AVQZ for HF method and PBE functionl on S22 database. The errors are in meV. 29

ST18 The minimum absolute error of Hard, Hard_GW, Standard, Standard_GW and Soft as well as AVDZ, AVTZ and AVQZ for HF method and PBE functionl on S22 database. The errors are in meV. 29

ST19 The minimum absolute error of Hard, Hard_GW, Standard, Standard_GW and Soft as well as AVDZ, AVTZ and AVQZ for HF method and PBE functionl on S66 database. The errors are in meV. 30

ST20 The coefficients of the fitting ($\mathrm{E_{int}^{err} = ae^{-bR} + cR^{-3}}$) equation of Hard potential error for the hydrogen bonded sample complexes. 30

ST21 The coefficients of the fitting ($\mathrm{E_{int}^{err} = ae^{-bR} + cR^{-3}}$) equation of Hard_*GW* potential error for the hydrogen bonded sample complexes. 30

ST22 The coefficients of the fitting ($\mathrm{E_{int}^{err} = ae^{-bR} + cR^{-3}}$) equation of Standard potential error for the hydrogen bonded sample complexes. 31

ST23 The coefficients of the fitting ($\mathrm{E_{int}^{err} = ae^{-bR} + cR^{-3}}$) equation of Standard_*GW* potential error for the hydrogen bonded sample complexes. 31

ST24 The coefficients of the fitting ($\mathrm{E_{int}^{err} = ae^{-bR} + cR^{-3}}$) equation of Soft potential error for the hydrogen bonded sample complexes. 31

ST25 The coefficients of the fitting ($\mathrm{E_{int}^{err} = ae^{-bR} + cR^{-3}}$) equation of Hard potential error for the direct oxygen-oxygen contact sample complexes. 32

ST26 The coefficients of the fitting ($\mathrm{E_{int}^{err} = ae^{-bR} + cR^{-3}}$) equation of Hard_*GW* potential error for the direct oxygen-oxygen contact sample complexes. 32

ST27 The coefficients of the fitting ($\mathrm{E_{int}^{err} = ae^{-bR} + cR^{-3}}$) equation of Standard potential error for the direct oxygen-oxygen contact sample complexes. 32

ST28 The coefficients of the fitting ($E_{int}^{err} = ae^{-bR} + cR^{-3}$) equation of Standard_GW potential error for the direct oxygen-oxygen contact sample complexes. 33

ST29 The coefficients of the fitting ($E_{int}^{err} = ae^{-bR} + cR^{-3}$) equation of Soft potential error for the direct oxygen-oxygen contact sample complexes. 33

ST30 The PBE interaction energies for S66 datasets for Hard, Hard_GW, Standard, Standard_GW, Soft, pslibrary1.0.0 (precision), pslibrary1.0.0 (efficiency) potentials as well as AVDZ, AVTZ, AVQZ and AV5Z basis sets. The Energies are in eV. 34

ST31 The HF interaction energies for S66 datasets for Hard, Hard_GW, Standard, Standard_GW, Soft, as well as AVDZ, AVTZ, AVQZ and AV5Z basis sets. The Energies are in eV. 36

ST32 The PBE interaction energies for S22 datasets for Hard, Hard_GW, Standard, Standard_GW, Soft, as well as AVDZ, AVTZ, AVQZ and AV5Z basis sets. The Energies are in eV. 38

ST33 The HF interaction energies for S22 datasets for Hard, Hard_GW, Standard, Standard_GW, Soft, as well as AVDZ, AVTZ, AVQZ and AV5Z basis sets. The Energies are in eV. 39

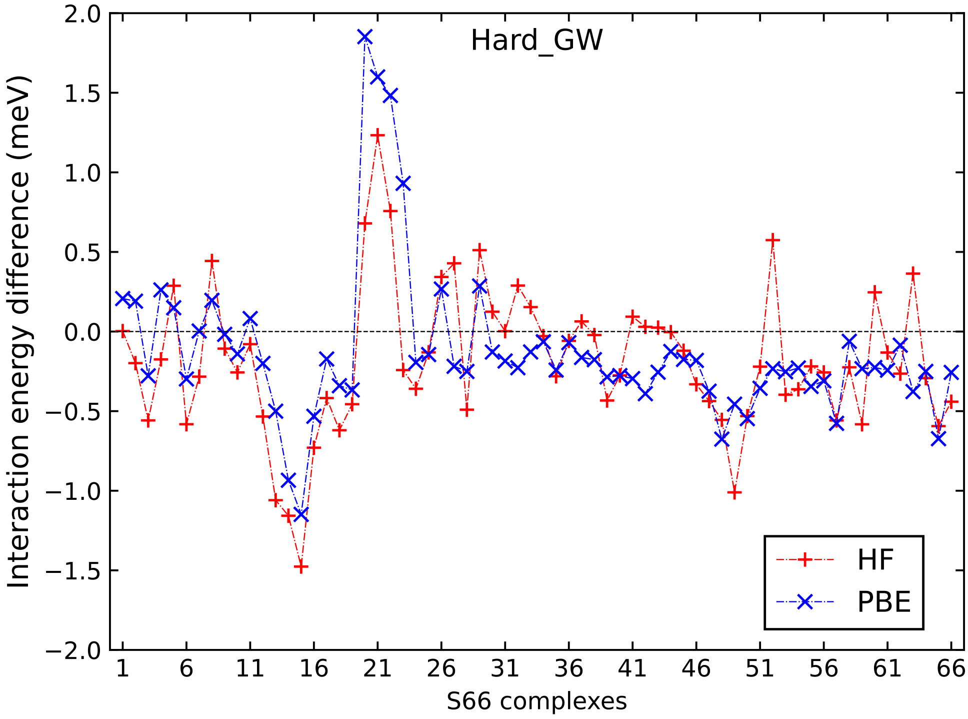

FIG. SF1: Variations in errors of Hard_GW potential across S66 dataset.

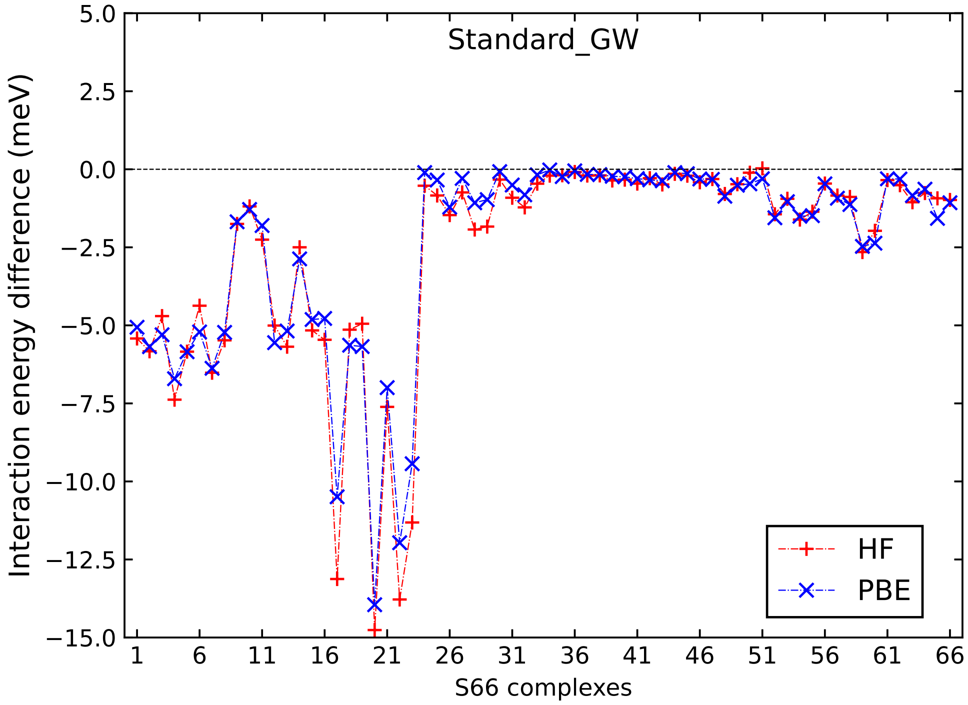

FIG. SF2: Variations in error of Standard_GW potential across S66 dataset.

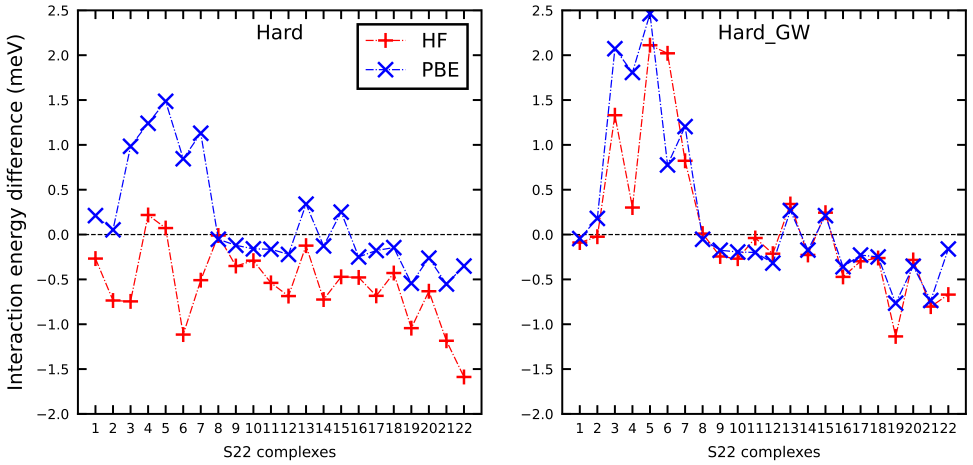

FIG. SF3: Variations of errors of Hard and Hard_GW potentials across S22 dataset.

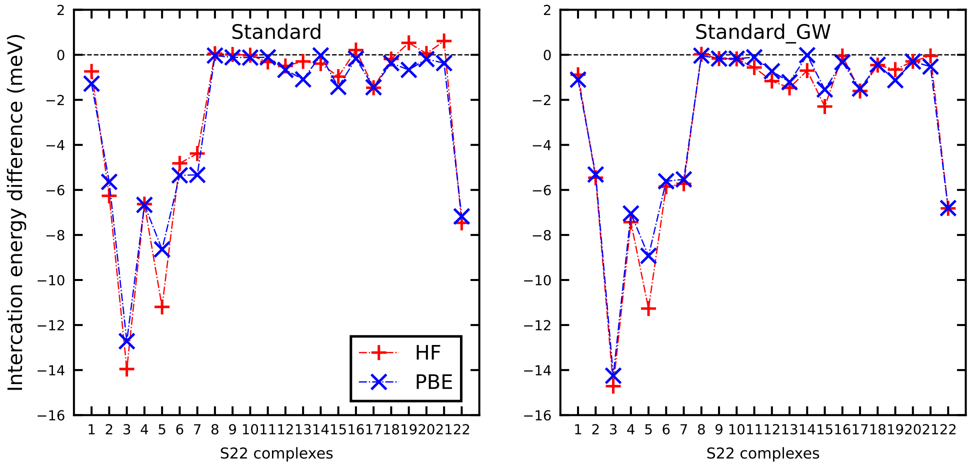

FIG. SF4: Variations of errors of Standard and Standard_GW potentials across S22 dataset.

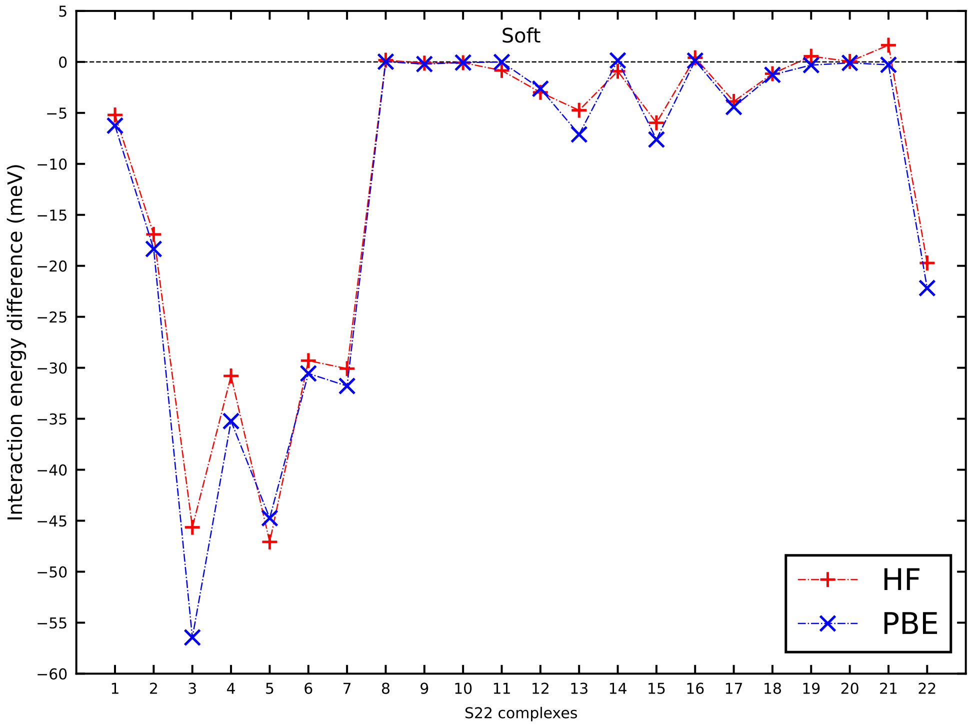

FIG. SF5: Variations of error of Soft potential across S22 dataset.

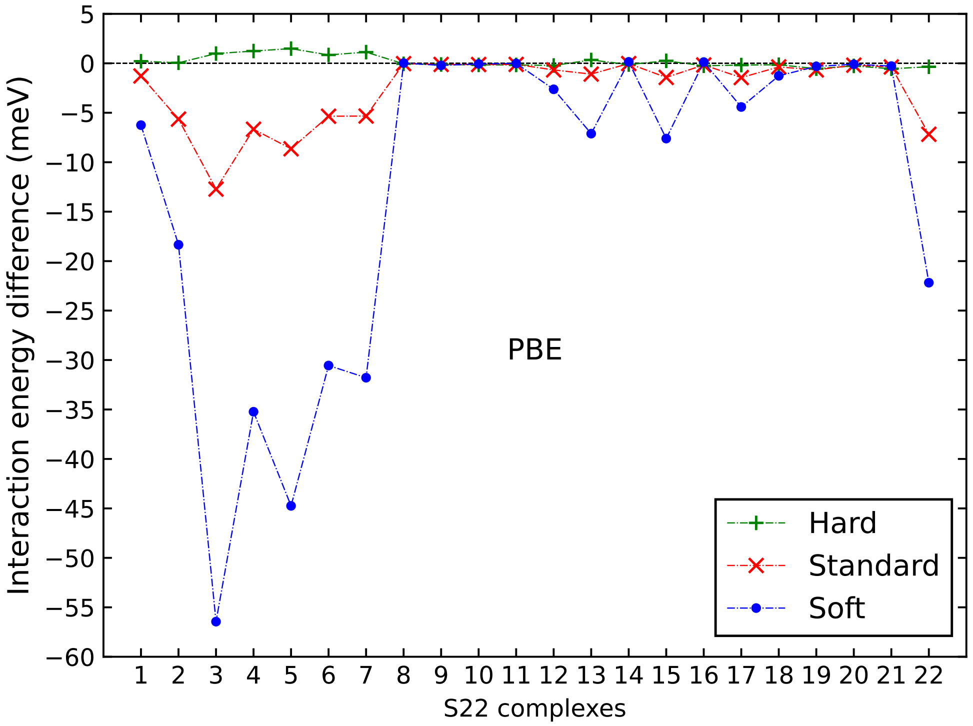

FIG. SF6: Variations in error of Hard, Standard and Soft potentials across S22 dataset for PBE functional.

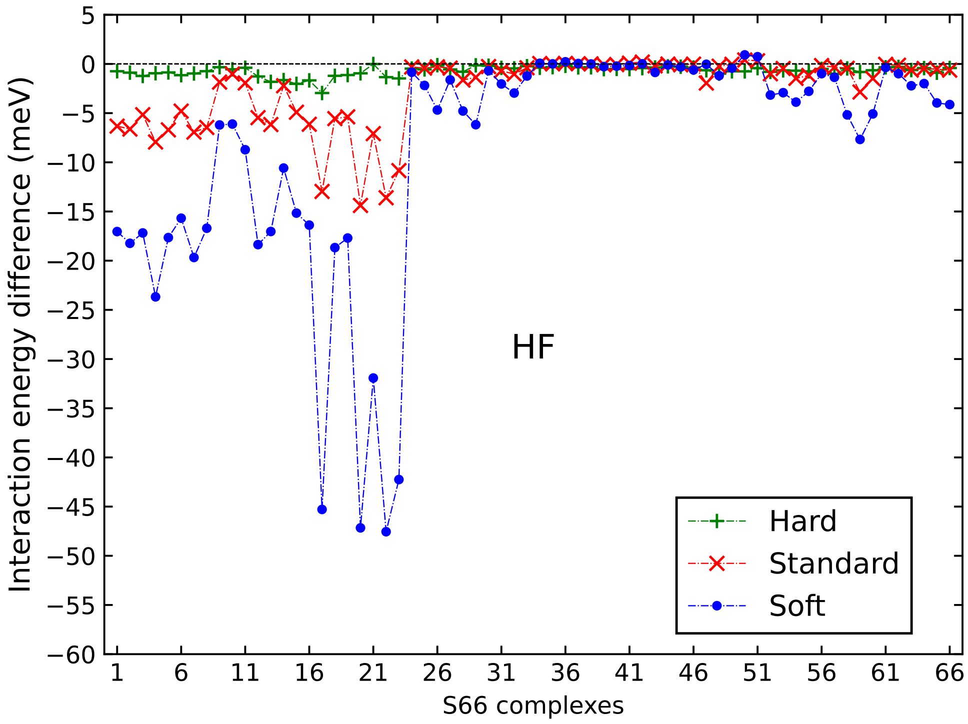

FIG. SF7: Variations in error of Hard, Standard and Soft potentials across S66 dataset for PBE method.

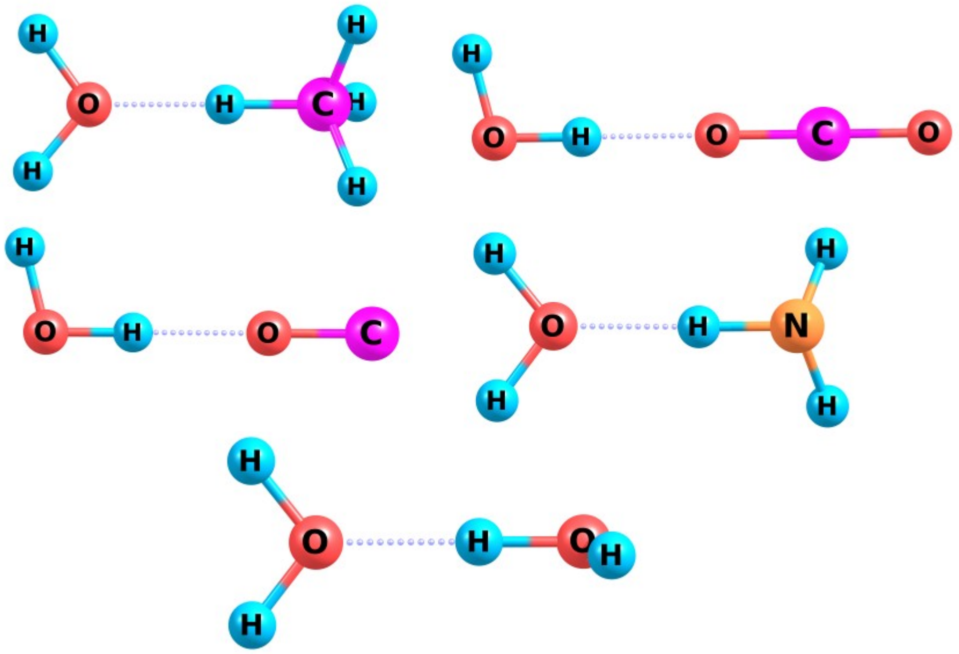

FIG. SF8: Hydrogen-bonded sample complexes.

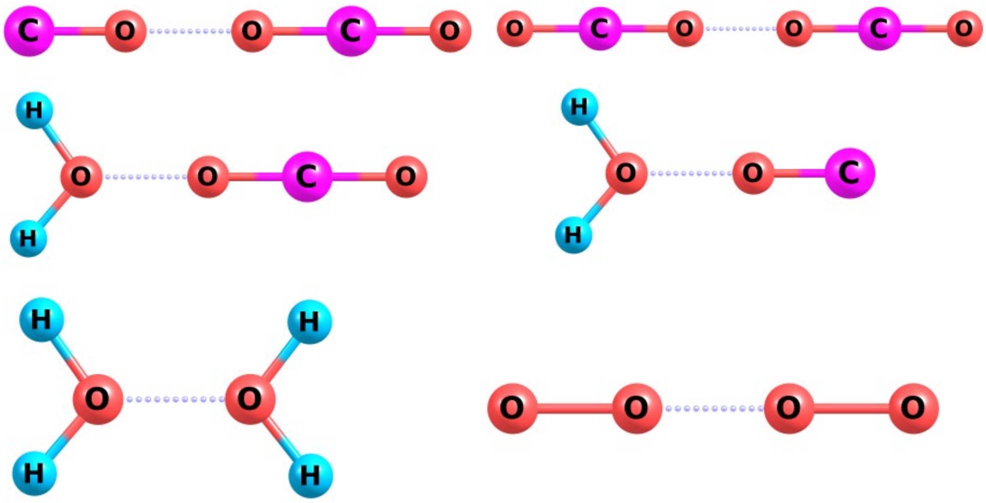

FIG. SF9: The O$\cdots$O direct contact complexes.

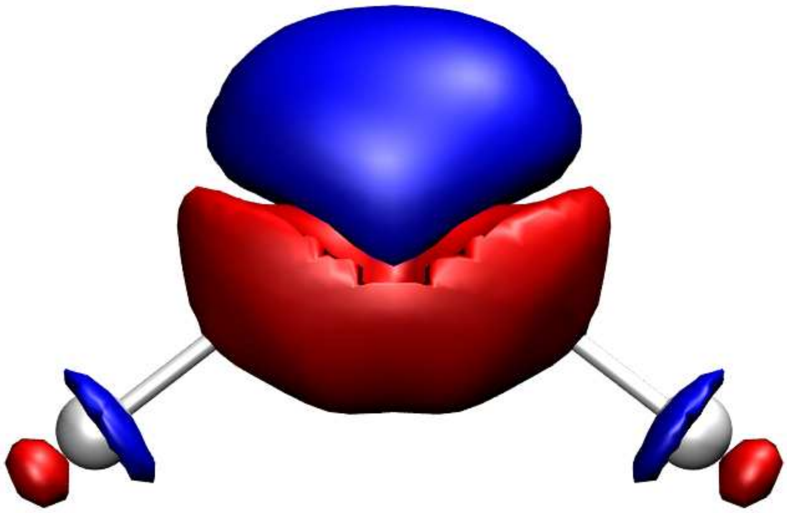

FIG. SF10: Electron density difference between Soft and Hard PAWs of water molecule. The red and blue iso-surfaces are plotted at values of -0.02 and 0.02 Å^{-3}.

TABLE ST1: The parameters of Hard potentials (`POTCAR`) used in the present study. Valence denotes which orbitals are considered as valence. r_c^l and r_{comp}^l refers to the cut-off radii of partial waves and radii of pseudo-partial-waves, respectively. Ecut† refers to the energy cut-off of the PAW potential. In all the cases two partial waves are used for the s and p orbitals.

Atom	Valence	r_c^l	r_{comp}^l	Ecut
H	$1s$	1.0	1.0	700
C	$2s2p$	1.1	1.1	700
N	$2s2p$	1.1	1.1	700
O	$2s2p$	1.1	1.1	700

† Ecut refers to the required energy cut-off to converge cohesive energie to a few meV, however, in the present study, a energy cut-off of 2000 eV has been used in all the calculation used Hard PAW potential.

TABLE ST2: The parameters of Hard_GW potentials (`POTCAR`) used in the present study. Valence denotes which orbitals are considered as valence. r_c^l and r_{comp}^l refers to the cut-off radii of partial waves and radii of pseudo-partial-waves, respectively. Ecut† refers to the energy cut-off of the PAW potential. In all the cases, but hydrogen, three partial waves are used for the s and p orbitals. For $1s$ of hydrogen atom only two partial waves are used.

Atom	Valence	r_c^l	r_{comp}^l	Ecut
H	$1s$	1.0	1.0	700
C	$2s2p$	1.1	1.1	741.689
N	$2s2p$	1.1	1.1	755.582
O	$2s2p$	1.1	1.1	765.519

† Ecut refers to the required energy cut-off to converge cohesive energie to a few meV, however, in the present study, a energy cut-off of 2000 eV has been used in all the calculation used Hard_GW PAW potential.

TABLE ST3: The parameters of Standard potentials (POTCAR) used in the present study. Valence denotes which orbitals are considered as valence. r_c^l and r_{comp}^l refers to the cut-off radii of partial waves and radii of pseudo-partial-waves, respectively. Ecut† refers to the energy cut-off of the PAW potential. In all the cases two partial waves are used for the s and p orbitals.

Atom	Valence	r_c^l	r_{comp}^l	Ecut
H	$1s$	1.1	1.1	250
C	$2s2p$	1.5	1.5	400
N	$2s2p$	1.5	1.5	400
O	$2s2p$	1.52	1.52	400

† Ecut refers to the required energy cut-off to converge cohesive energie to a few meV, however, in the present study, a energy cut-off of 1600 eV has been used in all the calculation used Standard PAW potential.

TABLE ST4: The parameters of Standard_GW potentials (POTCAR) used in the present study. Valence denotes which orbitals are considered as valence. r_c^l and r_{comp}^l refers to the cut-off radii of partial waves and radii of pseudo-partial-waves, respectively. Ecut† refers to the energy cut-off of the PAW potential. In all the cases two partial waves are used for the s and p orbitals.

Atom	Valence	r_c^l	r_{comp}^l	Ecut
H	$1s$	1.1	1.1	300
C	$2s2p$	1.5	1.5	413.992
N	$2s2p$	1.5	1.5	420.902
O	$2s2p$	1.52	1.52	414.635

† Ecut refers to the required energy cut-off to converge cohesive energie to a few meV, however, in the present study, a energy cut-off of 1600 eV has been used in all the calculation used Standard_GW PAW potential.

TABLE ST5: The parameters of Soft potentials (POTCAR) used in the present study. Valence denotes which orbitals are considered as valence. r_c^l and r_{comp}^l refers to the cut-off radii of partial waves and radii of pseudo-partial-waves, respectively. Ecut[†] refers to the energy cut-off of the PAW potential. In all the cases two partial waves are used for the s and p orbitals.

Atom	Valence	r_c^l	r_{comp}^l	Ecut
H	$1s$	1.3	1.3	200
C	$2s2p$	1.85	1.85	273.911
N	$2s2p$	1.85	1.85	279.692
O	$2s2p$	1.85	1.85	282.853

[†] Ecut refers to the required energy cut-off to converge cohesive energie to a few meV, however, in the present study, a energy cut-off of 1000 eV has been used in all the calculation used Soft PAW potential.

TABLE ST6: The parameters of pslibrary.1.0.0 PAW (precision) potentials used in the present study. Valence denotes which orbitals are considered as valence. r_{loc}, r_l, r_{core}, and r_{sph} refer to the radius of the local potential, the cut-off radii for the s, p, d orbitals, the pseudization radius of the core charge, and the values of radius of the PAW sphere respectively. Ecut[†] refers to the energy cut-off of the PAW potential (in eV).

Atom	Valence	r_{loc}(a.u)	r_l(a.u)		r_{core}(a.u)	r_{sph}($\mathring{A}$)	Ecut
			s	p			
H	$1s$	0.75	1.0	0.75		0.53	680.28
C	$2s2p$	0.9	1.2	1.4	0.7	0.75	544.22
N	$2s2p$	1.2	1.3	1.35	0.7	0.71	612.25
O	$2s2p$	1.1	1.3	1.35	0.7	0.71	680.28

TABLE ST7: The parameters of pslibrary.1.0.0 PAW (efficiency) potentials used in the present study. Valence denotes which orbitals are considered as valence. r_{loc}, r_l, r_{core}, and r_{sph} refer to the radius of the local potential, the cut-off radii for the s, p, d orbitals, the pseudization radius of the core charge, and the values of radius of the PAW sphere respectively. Ecut† refers to the energy cut-off of the PAW potential (in eV).

Atom	Valence	r_{loc}(a.u)	r_l(a.u)		r_{core}(a.u)	r_{sph}($\AA$)	Ecut
			s	p			
H	1s	0.75	1.0	0.75		0.53	680.28
C	2s2p	0.9	1.2	1.4	0.7	0.75	544.22
N	2s2p	1.2	1.3	1.35	0.7	0.71	612.25
O	2s2p	1.1	1.3	1.45	0.7	0.69	952.40

TABLE ST8: Errors of Hard, Hard_GW, Standard, Standard_GW and Soft potentials as well as AVDZ, AVTZ, and AVQZ with respect to AV5Z method on S66 data for PBE functional. The errors are in meV.

Number	Dimer	nature	category	Hard	Hard_GW	Standard	Standard_GW	Soft	AVDZ	AVTZ	AVQZ
1	Water-Water	E	HB	0.0532	0.2073	−5.5133	−5.0613	−17.8112	2.6937	1.7448	0.1148
2	Water-MeOH	E	HB	0.1092	0.1908	−5.9979	−5.683	−19.8025	1.5113	1.9162	0.1453
3	Water-MeNH2	E	HB	−0.0408	−0.2782	−5.3849	−5.3018	−20.2367	0.9812	0.8082	0.0207
4	Water-Peptide	E	HB	0.0957	0.2614	−6.7404	−6.7086	−24.9551	3.9857	2.0458	0.142
5	MeOH-MeOH	E	HB	−0.0038	0.1496	−6.2504	−5.8455	−19.7695	2.402	1.9355	0.1674
6	MeOH-MeNH2	E	HB	−0.0965	−0.2979	−5.3257	−5.2117	−19.4506	1.3769	0.6569	0.0863
7	MeOH-Peptide	E	HB	−0.1647	0.0034	−6.4031	−6.3754	−22.6564	4.7653	2.0384	0.0814
8	MeOH-Water	E	HB	−0.0289	0.1959	−5.7719	−5.2261	−17.9529	3.4795	1.7595	0.1488
9	MeNH2-MeOH	M	HB	0.0265	−0.0176	−1.8743	−1.6825	−6.5548	0.7938	0.6762	0.1184
10	MeNH2-MeNH2	M	HB	0.1537	−0.1404	−1.4291	−1.2878	−6.7849	1.2928	0.5355	0.0844
11	MeNH2-Peptide	M	HB	0.2415	0.0816	−1.827	−1.8023	−8.6628	1.572	0.6648	0.0656
12	MeNH2-Water	E	HB	0.0649	−0.2001	−5.6296	−5.5542	−21.4413	0.9747	0.8833	0.1459
13	Peptide-MeOH	E	HB	−0.6542	−0.5002	−5.698	−5.1813	−17.4524	3.335	1.2621	0.0933
14	Peptide-MeNH2	E	HB	−0.6412	−0.9341	−3.0128	−2.8703	−11.6451	0.8814	0.7393	0.086
15	Peptide-Peptide	E	HB	−1.2422	−1.1485	−4.8563	−4.8128	−16.4184	2.6303	1.4466	0.0071
16	Peptide-Water	E	HB	−0.7603	−0.5322	−5.3553	−4.7789	−16.0437	3.4431	1.0866	0.1336
17	Uracil-Uracil(BP)	E	HB	−0.9483	−0.1724	−10.3637	−10.4918	−43.975	6.8469	3.9824	0.1056
18	Water-Pyridine	E	HB	−0.1557	−0.3403	−5.6811	−5.6358	−21.8924	0.7173	1.0754	0.1214
19	MeOH-Pyridine	E	HB	−0.2342	−0.3668	−5.7726	−5.6792	−21.6457	1.4232	1.0944	0.1497
20	AcOH-AcOH	E	HB	0.881	1.8525	−12.681	−13.9498	−55.8747	12.6051	5.1609	0.3072
21	AcNH2-AcNH2	E	HB	1.0837	1.5994	−6.7507	−6.9953	−34.9096	4.7884	4.2627	0.0901
22	AcOH-Uracil	E	HB	0.5488	1.483	−11.2622	−11.9638	−50.5089	10.3844	4.4436	0.1951
23	AcNH2-Uracil	E	HB	0.1727	0.9309	−9.1214	−9.4327	−42.4284	6.1294	3.9353	0.102
24	Benzene-Benzene($\pi-\pi$)	D	$\pi-\pi$	−0.1675	−0.194	−0.1145	−0.1053	−0.0187	−1.7007	0.0136	−0.092
25	Pyridine-Pyridine($\pi-\pi$)	D	$\pi-\pi$	−0.0554	−0.1445	−0.3151	−0.3443	−1.4515	−1.7546	−0.0027	−0.1616
26	Uracil-Uracil($\pi-\pi$)	M	$\pi-\pi$	0.3389	0.2671	−1.0907	−1.2135	−7.1113	2.5462	−0.2863	−0.3927
27	Benzene-Pyridine($\pi-\pi$)	D	$\pi-\pi$	−0.1604	−0.2184	−0.287	−0.2936	−0.9013	−1.8613	0.0199	−0.1018
28	Benzene-Uracil($\pi-\pi$)	D	$\pi-\pi$	−0.2452	−0.2506	−1.1217	−1.0732	−3.8674	−0.1755	−0.0933	−0.1782
29	Pyridine-Uracil($\pi-\pi$)	D	$\pi-\pi$	0.3388	0.286	−0.9821	−0.972	−5.5236	−0.1303	−0.1053	−0.2629
30	Benzene-Ethene	D	$\pi-\pi$	−0.1395	−0.1287	−0.0992	−0.0755	−0.0541	−1.0389	0.0993	−0.1951
31	Uracil-Ethene	D	$\pi-\pi$	−0.1912	−0.185	−0.4679	−0.5047	−1.5794	0.5181	0.1518	−0.0773
32	Uracil-Ethyne	M	$\pi-\pi$	−0.2276	−0.2271	−0.7476	−0.8189	−2.4392	0.8819	0.0321	−0.2351
33	Pyridine-Ethene	D	$\pi-\pi$	−0.1275	−0.1281	−0.2068	−0.1783	−0.5907	−0.8433	0.1176	−0.2133
34	Pentane-Pentane	D	$\sigma-\sigma$	0.0111	−0.0642	0.053	−0.0221	0.0874	−1.8487	0.7178	−0.0044
35	Neopentane-Pentane	D	$\sigma-\sigma$	−0.1879	−0.2418	−0.1761	−0.2339	−0.1748	1.4202	0.7265	0.0139
36	Neopentane-Neopentane	D	$\sigma-\sigma$	−0.0402	−0.0699	−0.0085	−0.0498	0.0921	0.0231	0.5271	−0.0128
37	Cyclopentane-Neopentane	D	$\sigma-\sigma$	−0.1072	−0.1627	−0.092	−0.1649	−0.0583	0.6708	0.6196	−0.0683
38	Cyclopentane-Cyclopentane	D	$\sigma-\sigma$	−0.1216	−0.1766	−0.098	−0.1708	−0.0685	−0.0016	0.7135	−0.0365
39	Benzene-Cyclopentane	D	$\sigma-\pi$	−0.2177	−0.2852	−0.1551	−0.2243	−0.0523	−1.4678	0.1943	−0.0947
40	Benzene-Neopentane	D	$\sigma-\pi$	−0.2163	−0.2767	−0.1681	−0.2412	−0.0939	0.8811	0.0816	−0.178
41	Uracil-Pentane	D	$\sigma-\pi$	−0.218	−0.2957	−0.1926	−0.3002	−0.5935	0.6792	0.3981	−0.1325

Continued

TABLE ST8 − *continued*

Number	Dimer	nature	category	Hard	Hard_GW	Standard	Standard_GW	Soft	AVDZ	AVTZ	AVQZ
42	Uracil-Cyclopentane	D	$\sigma-\pi$	−0.3295	−0.3903	−0.253	−0.3401	−0.3933	0.6969	0.4106	−0.0699
43	Uracil-Neopentane	D	$\sigma-\pi$	−0.2262	−0.2554	−0.3244	−0.3684	−0.9643	−0.2242	0.1592	−0.0384
44	Ethene-Pentane	D	$\sigma-\pi$	−0.0814	−0.1254	−0.0719	−0.106	−0.1135	−1.1217	0.4596	0.055
45	Ethyne-Pentane	D	$\sigma-\pi$	−0.128	−0.175	−0.1119	−0.1418	−0.095	−1.6278	0.0776	−0.0672
46	Peptide-Pentane	D	$\sigma-\pi$	−0.0915	−0.1812	−0.2288	−0.3107	−0.9262	−0.0087	0.5108	0.0033
47	Benzene-Benzene(TS)	D	others	−0.2837	−0.3747	−0.2189	−0.324	−0.1462	0.664	0.1211	−0.1785
48	Pyridine-Pyridine(TS)	D	others	−0.5282	−0.6758	−0.7272	−0.864	−1.6969	0.2805	0.0756	−0.2509
49	Benzene-Pyridine(TS)	D	others	−0.3385	−0.4562	−0.3831	−0.5107	−0.7184	0.9323	0.058	−0.1927
50	Benzene-Ethyne(CH-π)	M	others	−0.3962	−0.547	−0.2469	−0.4663	0.211	3.5952	−0.2838	−0.2065
51	Ethyne-Ethyne(TS)	M	others	−0.2454	−0.3563	−0.1182	−0.3034	0.4208	1.5676	0.0675	−0.1105
52	Benzene-AcOH(OH-π)	M	others	−0.1843	−0.2336	−1.3437	−1.5536	−4.7615	6.2058	0.5777	0.1573
53	Benzene-AcNH2(NH-π)	M	others	−0.1367	−0.2524	−0.866	−1.0379	−3.8142	2.5813	0.4773	0.0035
54	Benzene-Water(OH-π)	M	others	−0.1774	−0.2288	−1.4444	−1.5054	−4.4243	2.0828	0.3703	0.086
55	Benzene-MeOH(OH-π)	M	others	−0.2831	−0.3447	−1.4483	−1.4861	−3.8928	3.7201	0.3249	−0.0125
56	Benzene-MeNH2(NH-π)	D	others	−0.1972	−0.3097	−0.4041	−0.4668	−1.2226	2.5628	0.1962	−0.1173
57	Benzene-Peptide(NH-π)	D	others	−0.454	−0.5767	−0.7987	−0.927	−2.1009	3.7035	0.0479	−0.1682
58	Pyridine-Pyridine(CH-N)	M	others	0.261	−0.0615	−1.0965	−1.1285	−5.9749	−0.6637	0.3029	0.0759
59	Ethyne-Water(CH-O)	E	others	−0.2542	−0.2332	−2.5876	−2.4699	−7.246	2.2561	0.5127	0.0634
60	Ethyne-AcOH(OH-π)	E	others	−0.181	−0.2257	−1.9323	−2.3683	−7.0085	6.0739	0.8136	0.1755
61	Pentane-AcOH	D	others	−0.1925	−0.2428	−0.2406	−0.3048	−0.6582	−0.0076	0.4825	0.117
62	Pentane-AcNH2	D	others	−0.0162	−0.0858	−0.2354	−0.3131	−1.1861	0.563	0.6144	0.0272
63	Benzene-AcOH	D	others	−0.3127	−0.377	−0.7206	−0.8401	−2.1964	−0.0079	0.1614	−0.0882
64	Peptide-Ethene	D	others	−0.1859	−0.2504	−0.5596	−0.6372	−2.0193	0.7138	0.3018	−0.0751
65	Pyridine-Ethyne	E	others	−0.349	−0.6729	−1.3549	−1.5724	−4.5659	−0.086	0.3075	0.0054
66	MeNH2-Pyridine	M	others	−0.0671	−0.2567	−1.1088	−1.0723	−4.8892	0.1984	0.3812	−0.0773

TABLE ST9: The maximum absolute error of Hard, Hard_GW, Standard, Standard_GW and Soft as well as AVDZ, AVTZ and AVQZ for HF method and PBE functionl on S66 database. The errors are in meV.

	PBE					HF				
potential/basis set	number	complex	type	nature	max	number	complex	type	nature	max
Hard	15	Peptide-Peptide	HB	E	1.2422	17	Uracil-Uracil(BP)	HB	E	2.9554
Hard_GW	20	AcOH-AcOH	HB	E	1.8525	15	Peptide-Peptide	HB	E	1.4762
Standard	20	AcOH-AcOH	HB	E	12.681	20	AcOH-AcOH	HB	E	14.3852
Standard_GW	20	AcOH-AcOH	HB	E	13.9498	20	AcOH-AcOH	HB	E	14.7607
Soft	20	AcOH-AcOH	HB	E	55.8747	22	AcOH-Uracil	HB	E	47.5513
AVDZ	20	AcOH-AcOH	HB	E	12.6051	20	AcOH-AcOH	HB	E	17.2354
AVTZ	20	AcOH-AcOH	HB	E	5.1609	20	AcOH-AcOH	HB	E	4.5653
AVQZ	26	Uracil-Uracil(π-π)	$\pi - \pi$	M	0.3927	20	AcOH-AcOH	HB	E	0.3513

TABLE ST10: Errors of Hard, Hard_GW, Standard, Standard_GW and Soft potentials as well as AVDZ, AVTZ, and AVQZ with respect to AV5Z method on S22 data for PBE functional. The errors are in meV.

Number	Dimer	Hard	Hard_GW	Standard	Standard_GW	Soft	AVDZ	AVTZ	AVQZ
1	Ammonia dimer	0.2113	−0.0448	−1.2814	−1.1027	−6.2511	3.1195	0.1527	0.0318
2	Water dimer	0.0526	0.1800	−5.6425	−5.3156	−18.3457	2.6215	1.8128	0.0947
3	Formic acid dimer	0.9840	2.0724	−12.7182	−14.2507	−56.4479	15.4343	5.2102	0.2939
4	Formamide dimer	1.2406	1.8076	−6.6646	−7.0463	−35.2288	7.1362	4.2689	0.0977
5	Uracil dimer	1.4855	2.4626	−8.6398	−8.9273	−44.7439	8.3259	3.7231	0.1167
6	2-pyridoxine-2-aminopyridine	0.8455	0.7760	−5.3515	−5.6147	−30.5547	4.6376	2.8452	0.1927
7	Adenine-thymine	1.1297	1.2040	−5.3310	−5.5301	−31.7864	4.7073	3.0716	0.1984
8	Methane dimer	−0.0525	−0.0525	−0.0332	−0.0381	0.0227	−0.2495	−0.2139	0.0503
9	Ethene dimer	−0.1198	−0.1751	−0.1134	−0.1667	−0.2099	−0.3050	0.3456	−0.0210
10	Benzene-methane	−0.1596	−0.1942	−0.1221	−0.1700	−0.0630	1.9532	0.2985	−0.1804
11	Benzene dimer(stack)	−0.1636	−0.1997	−0.1094	−0.1079	−0.0119	−2.0917	0.0735	−0.0424
12	Pyrazine dimer	−0.2214	−0.3179	−0.6695	−0.7281	−2.6260	−0.4748	0.0664	−0.2969
13	Uracil dimer(stack)	0.3390	0.2673	−1.0905	−1.2133	−7.1112	2.5464	−0.2863	−0.3927
14	Indole-benzene(stack)	−0.1269	−0.1728	−0.0277	−0.0176	0.1423	−3.5473	0.1235	−0.0112
15	Adenine-thymine(stack)	0.2504	0.2108	−1.4256	−1.5386	−7.6089	−1.4506	0.1208	−0.2218
16	Ethene-ethyne	−0.2498	−0.3591	−0.1697	−0.3299	0.1251	1.6768	−0.1336	−0.1108
17	Benzene-water	−0.1777	−0.2286	−1.4414	−1.5015	−4.4154	2.0550	0.3665	0.0857

Continued

TABLE ST10 − ***continued***

Number	Dimer	Hard	Hard_GW	Standard	Standard_GW	Soft	AVDZ	AVTZ	AVQZ
18	Benzene-ammonia	−0.1455	−0.2495	−0.3674	−0.4397	−1.2653	2.5415	0.3823	−0.0509
19	Benzene-HCN	−0.5426	−0.7653	−0.6688	−1.1281	−0.3103	6.3541	−0.6365	−0.3097
20	Benzene dimer(TS)	−0.2628	−0.3529	−0.1886	−0.2972	−0.0954	2.7709	0.2114	−0.1094
21	Indolebenzene(TS)	−0.5516	−0.7346	−0.3679	−0.5241	−0.2823	6.6902	0.1603	−0.1135
22	Phenol dimer	−0.3511	−0.1610	−7.1760	−6.8067	−22.1807	3.2050	1.7413	0.0014

TABLE ST11: Root mean square error (RMSE), mean absolute error (MAE) and average error (AVG) of Hard, Hard_GW, Standard, Standard_GW and Soft potentials as well as AVDZ, AVTZ, and AVQZ basis sets with respect to AV5Z basis set on S22 data for PBE functional. The errors are in meV.

potential/basis set	RMSE	MAE	AVG
Hard	0.6019	0.4393	0.1552
Hard_GW	0.8942	0.5904	0.226
Standard	4.418	2.7091	−2.7091
Standard_GW	4.666	2.8543	−2.8543
Soft	20.6461	12.2649	−12.2386
AVDZ	5.068	3.8134	3.0753
AVTZ	1.9569	1.1929	1.0775
AVQZ	0.1742	0.1374	−0.0317

TABLE ST12: Errors of Hard, Hard_GW, Standard, Standard_GW and Soft potentials as well as AVDZ, AVTZ, and AVQZ with respect to AV5Z method on S66 data for HF method. The errors are in meV.

Number	Dimer	nature	category	Hard	Hard_GW	Standard	Standard_GW	Soft	AVDZ	AVTZ	AVQZ
1	Water-Water	E	HB	−0.7418	0.0029	−6.3223	−5.4208	−17.0406	0.5431	1.467	−0.0144
2	Water-MeOH	E	HB	−0.884	−0.1985	−6.6217	−5.8303	−18.2343	−0.2735	1.6131	−0.0479
3	Water-MeNH2	E	HB	−1.2234	−0.5582	−5.1605	−4.7032	−17.1816	0.0033	0.5921	−0.0073
4	Water-Peptide	E	HB	−0.945	−0.1749	−7.933	−7.3805	−23.6773	2.5418	1.4213	0.0052
5	MeOH-MeOH	E	HB	−0.8637	0.2877	−6.7018	−5.8428	−17.6512	0.9108	1.7954	0.0125
6	MeOH-MeNH2	E	HB	−1.1434	−0.5822	−4.7946	−4.3709	−15.6831	0.3693	0.7652	0.0498
7	MeOH-Peptide	E	HB	−0.9695	−0.2831	−6.9301	−6.5176	−19.6752	2.8518	1.9434	0.0721
8	MeOH-Water	E	HB	−0.7337	0.4434	−6.4555	−5.4794	−16.7009	1.6324	1.5829	0.034
9	MeNH2-MeOH	M	HB	−0.3287	−0.1071	−1.8454	−1.7505	−6.2026	−0.8275	0.7184	0.0128
10	MeNH2-MeNH2	M	HB	−0.5439	−0.2566	−1.0248	−1.1894	−6.1123	0.6972	0.4985	0.0079
11	MeNH2-Peptide	M	HB	−0.3874	−0.0793	−1.9343	−2.2542	−8.7211	0.0661	0.8169	0.0457
12	MeNH2-Water	E	HB	−1.2718	−0.5337	−5.459	−5.0063	−18.3658	0.034	0.4996	0.0003
13	Peptide-MeOH	E	HB	−1.8191	−1.0595	−6.1591	−5.6842	−17.0318	1.4199	1.0147	0.0147
14	Peptide-MeNH2	E	HB	−1.6413	−1.1569	−2.2158	−2.4993	−10.5842	−0.947	0.4925	0.0218
15	Peptide-Peptide	E	HB	−2.0241	−1.4762	−4.9075	−5.162	−15.163	1.1266	1.381	0.0307
16	Peptide-Water	E	HB	−1.672	−0.7297	−6.1333	−5.4626	−16.3831	1.5358	0.858	0.0316
17	Uracil-Uracil(BP)	E	HB	−2.9554	−0.4179	−12.9608	−13.1256	−45.2887	9.0965	3.6139	0.0901
18	Water-Pyridine	E	HB	−1.2079	−0.62	−5.5564	−5.1384	−18.6701	2.9478	0.6683	0.0199
19	MeOH-Pyridine	E	HB	−1.1288	−0.4564	−5.3768	−4.948	−17.6981	3.4197	0.7826	0.0664
20	AcOH-AcOH	E	HB	−0.9523	0.6794	−14.3852	−14.7607	−47.1611	17.2354	4.5653	0.3513
21	AcNH2-AcNH2	E	HB	−0.0083	1.2332	−7.0836	−7.6122	−31.9282	5.1805	3.7647	0.0776
22	AcOH-Uracil	E	HB	−1.3406	0.757	−13.6028	−13.7812	−47.5513	13.8462	3.9027	0.2269
23	AcNH2-Uracil	E	HB	−1.4723	−0.2421	−10.8435	−11.3149	−42.2498	7.3471	3.3965	0.1254
24	Benzene-Benzene($\pi-\pi$)	D	$\pi-\pi$	−0.473	−0.3593	−0.3053	−0.527	−0.8455	0.5192	0.1663	0.0596
25	Pyridine-Pyridine($\pi-\pi$)	D	$\pi-\pi$	−0.5561	−0.1309	−0.4221	−0.8397	−2.1858	−0.3584	0.1875	0.0528
26	Uracil-Uracil($\pi-\pi$)	M	$\pi-\pi$	−0.1236	0.3427	−0.2933	−1.4676	−4.6821	0.603	0.3695	0.08
27	Benzene-Pyridine($\pi-\pi$)	D	$\pi-\pi$	−0.5581	0.4287	−0.4238	−0.7414	−1.6155	−0.0879	0.1625	0.0561
28	Benzene-Uracil($\pi-\pi$)	D	$\pi-\pi$	−0.7942	−0.4909	−1.6369	−1.9295	−4.7848	0.9984	0.0367	0.0288
29	Pyridine-Uracil($\pi-\pi$)	D	$\pi-\pi$	−0.1423	0.5113	−1.3589	−1.8357	−6.1752	1.7301	0.0985	0.0438
30	Benzene-Ethene	D	$\pi-\pi$	−0.1802	0.1247	−0.2658	−0.3303	−0.6898	1.3157	0.1861	0.0395
31	Uracil-Ethene	D	$\pi-\pi$	−0.4003	0.0022	−0.6969	−0.9117	−2.0327	2.5538	0.1042	0.0264
32	Uracil-Ethyne	M	$\pi-\pi$	−0.4538	0.2889	−1.0323	−1.221	−2.9587	3.076	−0.0667	0.0141
33	Pyridine-Ethene	D	$\pi-\pi$	−0.2286	0.1535	−0.3906	−0.462	−1.2259	1.3135	0.2033	0.0185
34	Pentane-Pentane	D	$\sigma-\sigma$	−0.3833	−0.0287	0.0413	−0.2038	0.0664	0.3178	0.2016	0.049
35	Neopentane-Pentane	D	$\sigma-\sigma$	−0.3243	−0.2809	0.0076	−0.1918	0.0095	1.2678	0.1932	0.0411
36	Neopentane-Neopentane	D	$\sigma-\sigma$	−0.1895	−0.0587	0.0548	−0.0874	0.2183	0.7426	0.1061	0.0343
37	Cyclopentane-Neopentane	D	$\sigma-\sigma$	−0.3467	0.0637	0.0165	−0.2012	0.0443	0.917	0.1888	0.0479
38	Cyclopentane-Cyclopentane	D	$\sigma-\sigma$	−0.3393	−0.022	0.0077	−0.1964	−0.0037	0.5431	0.1766	0.0487
39	Benzene-Cyclopentane	D	$\sigma-\pi$	−0.5257	−0.4331	−0.0873	−0.3589	−0.2774	0.345	0.1916	0.0207
40	Benzene-Neopentane	D	$\sigma-\pi$	−0.5038	−0.2755	−0.065	−0.3323	−0.2222	0.6721	0.1546	0.0101
41	Uracil-Pentane	D	$\sigma-\pi$	−0.5224	0.0934	0.0805	−0.4588	−0.1596	1.2324	0.1826	0.0762

Continued

TABLE ST12 – *continued*

Number	Dimer	nature	category	Hard	Hard_GW	Standard	Standard_GW	Soft	AVDZ	AVTZ	AVQZ
42	Uracil-Cyclopentane	D	$\sigma - \pi$	−0.4244	0.0297	0.1881	−0.2858	0.0092	0.9597	0.188	0.0566
43	Uracil-Neopentane	D	$\sigma - \pi$	−0.3607	0.0247	−0.197	−0.4862	−0.8617	1.0988	0.1238	0.0498
44	Ethene-Pentane	D	$\sigma - \pi$	−0.2429	−0.0037	−0.0112	−0.1462	−0.1063	0.1347	0.1309	0.0248
45	Ethyne-Pentane	D	$\sigma - \pi$	−0.2793	−0.1204	−0.0884	−0.2054	−0.3097	−0.3004	0.0893	0.0218
46	Peptide-Pentane	D	$\sigma - \pi$	−0.3714	−0.3313	−0.0211	−0.4162	−0.6093	1.1301	0.2449	0.0637
47	Benzene-Benzene(TS)	D	others	−0.6419	−0.4369	−1.9454	−0.3127	−0.035	−0.9173	−0.0019	−0.0212
48	Pyridine-Pyridine(TS)	D	others	−0.9894	−0.5549	−0.2298	−0.7916	−1.1902	−2.3464	0.0863	−0.0068
49	Benzene-Pyridine(TS)	D	others	−0.7493	−1.0102	−0.0338	−0.4789	−0.4245	−0.9905	−0.0294	−0.0226
50	Benzene-Ethyne(CH-π)	M	others	−0.756	−0.5312	0.4049	−0.1101	0.9211	1.0022	−0.3001	−0.0577
51	Ethyne-Ethyne(TS)	M	others	−0.4223	−0.2204	0.3209	0.0295	0.7368	1.7312	0.0961	−0.0294
52	Benzene-AcOH(OH-π)	M	others	−0.8084	0.5743	−0.9963	−1.4409	−3.1631	2.7696	−0.1393	0.0019
53	Benzene-AcNH2(NH-π)	M	others	−0.6537	−0.3968	−0.4616	−0.9452	−2.9225	−2.6844	−0.0631	0.0283
54	Benzene-Water(OH-π)	M	others	−0.6859	−0.3626	−1.4638	−1.6116	−3.8815	−1.6743	0.0933	−0.0158
55	Benzene-MeOH(OH-π)	M	others	−0.7549	−0.2187	−1.1716	−1.3587	−2.7822	0.2871	0.068	0.0065
56	Benzene-MeNH2(NH-π)	D	others	−0.5084	−0.2569	−0.1635	−0.4571	−0.9867	0.5695	0.0384	−0.0136
57	Benzene-Peptide(NH-π)	D	others	−1.0082	−0.5584	−0.328	−0.8419	−1.3608	0.258	−0.0884	−0.0071
58	Pyridine-Pyridine(CH-N)	M	others	−0.455	−0.2245	−0.4402	−0.8861	−5.1785	−0.4082	0.135	0.0125
59	Ethyne-Water(CH-O)	E	others	−0.8195	−0.5826	−2.8536	−2.6512	−7.6745	0.6708	0.4985	0.0395
60	Ethyne-AcOH(OH-π)	E	others	−0.6428	0.2464	−1.4806	−1.9711	−5.0783	6.3247	0.1513	0.1461
61	Pentane-AcOH	D	others	−0.3417	−0.1317	−0.037	−0.3464	−0.3572	0.4302	0.1429	0.059
62	Pentane-AcNH2	D	others	−0.3031	−0.2644	−0.1325	−0.51	−0.9898	0.7557	0.1997	0.0697
63	Benzene-AcOH	D	others	−0.6197	0.3641	−0.6242	−1.0559	−2.219	−1.1178	0.166	0
64	Peptide-Ethene	D	others	−0.4196	−0.2928	−0.4638	−0.7611	−1.9966	−0.4253	0.1937	0.0201
65	Pyridine-Ethyne	E	others	−0.894	−0.5941	−0.5331	−0.9307	−3.9634	0.3559	0.2449	−0.0256
66	MeNH2-Pyridine	M	others	−0.4126	−0.4407	−0.6294	−0.9831	−4.1292	−0.7897	0.4713	0.0147

TABLE ST13: Errors of Hard, Hard_GW, Standard, Standard_GW and Soft potentials as well as AVDZ, AVTZ, and AVQZ with respect to AV5Z method on S22 data for HF method. The errors are in meV.

Number	Dimer	Hard	Hard_GW	Standard	Standard_GW	Soft	AVDZ	AVTZ	AVQZ
1	Ammonia dimer	−0.2678	−0.089	−0.7374	−0.8829	−5.201	1.551	0.2966	0.0256
2	Water dimer	−0.7353	−0.0241	−6.2612	−5.457	−16.9139	0.7121	1.5638	−0.0218
3	Formic acid dimer	−0.7452	1.3307	−13.9528	−14.7152	−45.6428	20.245	4.5353	0.4101
4	Formamide dimer	0.2182	0.3013	−6.6302	−7.4309	−30.7985	6.5498	3.8205	0.1306
5	Uracil dimer	0.0724	2.1114	−11.195	−11.2676	−47.0766	10.7627	3.2605	0.1508
6	2-pyridoxine-2-aminopyridine	−1.1151	2.0211	−4.8192	−5.8542	−29.2976	7.6121	2.3396	0.1328
7	Adenine-thymine	−0.5086	0.8227	−4.3821	−5.7268	−30.0817	6.9892	2.3021	0.1497
8	Methane dimer	−0.0106	0.0106	0.053	0.0232	0.1697	−0.2757	0.0356	0.0185
9	Ethene dimer	−0.3507	−0.2445	0.0153	−0.1679	−0.1154	0.0833	0.0544	0.0035
10	Benzene-methane	−0.2911	−0.2689	−0.0168	−0.1812	−0.0774	0.6517	0.1518	−0.015
11	Benzene dimer(stack)	−0.5388	−0.0387	−0.3169	−0.5627	−0.839	0.0286	0.1516	0.0629
12	Pyrazine dimer	−0.6859	−0.2133	−0.4953	−1.1657	−2.9862	0.4838	0.3137	0.0604
13	Uracil dimer(stack)	−0.1235	0.3406	−0.2931	−1.4673	−4.7429	0.603	0.3695	0.0803
14	Indole-benzene(stack)	−0.7246	−0.2274	−0.391	−0.7011	−0.912	0.8536	0.1097	0.0748
15	Adenine-thymine(stack)	−0.4713	0.2439	−0.9675	−2.2958	−5.9721	1.2667	0.2914	0.095
16	Ethene-ethyne	−0.478	−0.4721	0.2116	−0.0504	0.4069	0.6953	−0.0903	−0.0495
17	Benzene-water	−0.6834	−0.2977	−1.4612	−1.6072	−3.8767	−1.7037	0.0933	−0.0158
18	Benzene-ammonia	−0.4288	−0.2618	−0.186	−0.4555	−1.1567	0.218	0.1407	−0.0316
19	Benzene-HCN	−1.0435	−1.1358	0.5315	−0.6556	0.5591	1.7399	−0.3902	−0.0604
20	Benzene dimer(TS)	−0.6321	−0.2831	0.0592	−0.2908	0.0467	−0.04	−0.135	−0.0207
21	Indolebenzene(TS)	−1.1842	−0.8035	0.6127	−0.0485	1.6444	1.2425	−0.3842	−0.0177
22	Phenol dimer	−1.5881	−0.6695	−7.4621	−6.818	−19.7197	0.9761	1.4681	0.0482

TABLE ST14: Root mean square error (RMSE), mean absolute error (MAE) and average error (AVG) of Hard, Hard_GW, Standard, Standard_GW and Soft potentials as well as AVDZ, AVTZ, and AVQZ basis sets with respect to AV5Z basis set on S22 data for HF method. The errors are in meV.

potential/basis set	RMSE	MAE	AVG
Hard	0.6989	0.5862	−0.5598
Hard_GW	0.8074	0.5551	0.0979
Standard	4.7969	2.7751	−2.6402
Standard_GW	5.0263	3.083	−3.0809
Soft	18.8312	11.2835	−11.0265
AVDZ	5.6032	2.9674	2.7839
AVTZ	1.6787	1.0136	0.9227
AVQZ	0.1146	0.0762	0.055

TABLE ST15: Root mean square error (RMSE), mean absolute error (MAE) and average error (AVG) of Hard, Hard_GW, Standard, Standard_GW and Soft potentials as well as AVDZ, AVTZ, and AVQZ basis sets with respect to AV5Z basis set on S66 data for PBE functional for five types of complexes based on the geometrical orientations of monomers: hydrogen bonded (HB), aromatic-aromatic ($\pi-\pi$), aromatic-aliphatic ($\pi-\sigma$), aliphatic-aliphatic ($\sigma-\sigma$) and others. The errors are in meV.

	HB			$\pi-\pi$			$\pi-\sigma$			$\sigma-\sigma$			others		
potential/basis set	RMSE	MAE	AVG	RMSE	MAE	AVG	RMSE	MAE	AVG	RMSE	MAE	AVG	RMSE	MAE	AVG
Hard	0.5263	0.3653	−0.0669	0.2169	0.1992	−0.0637	0.204	0.1886	−0.1886	0.1125	0.0936	−0.0892	0.2795	0.2522	-0.2261
Hard_GW	0.7373	0.5167	0.0881	0.21	0.2029	−0.0923	0.2603	0.2481	−0.2481	0.1582	0.143	−0.143	0.3774	0.3381	-0.3381
Standard	6.6147	6.0306	−6.0306	0.6661	0.5433	−0.5433	0.2027	0.1882	−0.1882	0.102	0.0855	−0.0643	1.095	0.8918	-0.8918
Standard_GW	6.6911	5.9796	−5.9796	0.689	0.5579	−0.5579	0.2688	0.2541	−0.2541	0.151	0.1283	−0.1283	1.1911	1.0076	-1.0076
Soft	26.793	23.4292	−23.4292	3.2826	2.3537	−2.3537	0.5395	0.404	−0.404	0.1047	0.0962	−0.0244	3.7035	2.9577	-2.8945
AVDZ	4.5774	3.4354	3.4354	1.3712	1.1451	−0.3558	0.9897	0.8384	−0.2741	1.0849	0.7929	0.0527	2.6805	1.9233	1.8468
AVTZ	2.3647	1.9198	1.9198	0.1225	0.0922	−0.0053	0.3307	0.2865	0.2865	0.6654	0.6609	0.6609	0.3833	0.3239	0.2955
AVQZ	0.1317	0.1179	0.1179	0.2109	0.191	−0.191	0.0949	0.0799	−0.0653	0.0357	0.0272	−0.0216	0.1295	0.1094	-0.0383

TABLE ST16: Root mean square error (RMSE), mean absolute error (MAE) and average error (AVG) of Hard, Hard_GW, Standard, Standard_GW and Soft potentials as well as AVDZ, AVTZ, and AVQZ basis sets with respect to AV5Z basis set on S66 data for HF functional for four types of complexes based on the geometrical orientations of monomers: hydrogen bonded (HB), aromatic-aromatic ($\pi-\pi$), aromatic-aliphatic ($\pi-\sigma$), aliphatic-aliphatic ($\sigma-\sigma$) and others. The errors are in meV.

	HB			$\pi-\pi$			$\pi-\sigma$			$\sigma-\sigma$			others		
potential/basis set	RMSE	MAE	AVG	RMSE	MAE	AVG	RMSE	MAE	AVG	RMSE	MAE	AVG	RMSE	MAE	AVG
Hard	1.2965	1.1417	−1.1417	0.4429	0.391	−0.391	0.4166	0.4038	−0.4038	0.3235	0.3166	−0.3166	0.6756	0.6443	−0.6443
Hard_GW	0.6595	0.5363	−0.2404	0.3275	0.2833	0.0871	0.223	0.164	−0.127	0.1325	0.0908	−0.0653	0.4581	0.4131	−0.2947
Standard	7.4045	6.5395	−6.5395	0.8269	0.6826	−0.6826	0.1123	0.0923	−0.0252	0.0319	0.0256	0.0256	1.0196	0.7357	−0.6631
Standard_GW	7.2601	6.3146	−6.3146	1.1586	1.0266	−1.0266	0.3544	0.3362	−0.3362	0.1817	0.1761	−0.1761	1.1159	0.9237	−0.9207
Soft	24.7549	21.5198	−21.5198	3.2545	2.7196	−2.7196	0.4143	0.3194	−0.3171	0.1041	0.0684	0.067	3.1737	2.4995	−2.3338
AVDZ	5.513	3.2545	3.0764	1.5556	1.2556	1.1663	0.8369	0.7342	0.6591	0.8241	0.7577	0.7577	1.911	1.3254	0.19
AVTZ	2.0717	1.6589	1.6589	0.1811	0.1581	0.1448	0.1694	0.1632	0.1632	0.1767	0.1733	0.1733	0.2062	0.1604	0.0982
AVQZ	0.0991	0.0594	0.0533	0.0463	0.042	0.042	0.0463	0.0404	0.0404	0.0446	0.0442	0.0442	0.0443	0.0299	0.0099

TABLE ST17: The maximum absolute error of Hard, Hard_GW, Standard, Standard_GW and Soft as well as AVDZ, AVTZ and AVQZ for HF method and PBE functionl on S22 database. The errors are in meV.

	PBE					HF				
potential/basis set	number	complex	type	nature	max	number	complex	type	nature	max
Hard	5	Uracil dimer	HB	E	1.4855	22	Phenol dimer	others	M	1.5881
Hard_GW	5	Uracil dimer	HB	E	2.4626	5	Uracil dimer	HB	E	2.1114
Standard	3	Formic acid dimer	HB	E	12.7182	3	Formic acid dimer	HB	E	13.9528
Standard_GW	3	Formic acid dimer	HB	E	14.2507	3	Formic acid dimer	HB	E	14.7152
Soft	3	Formic acid dimer	HB	E	56.4479	5	Uracil dimer	HB	E	47.0766
AVDZ	3	Formic acid dimer	HB	E	15.4343	3	Formic acid dimer	HB	E	20.245
AVTZ	3	Formic acid dimer	HB	E	5.2102	3	Formic acid dimer	HB	E	4.5353
AVQZ	13	Uracil dimer(stack)	$\pi-\pi$	M	0.3927	3	Formic acid dimer	HB	E	0.4101

TABLE ST18: The minimum absolute error of Hard, Hard_GW, Standard, Standard_GW and Soft as well as AVDZ, AVTZ and AVQZ for HF method and PBE functionl on S22 database. The errors are in meV.

	PBE					HF				
potential/basis set	number	complex	type	nature	min	number	complex	type	nature	min
Hard	8	Methane dimer	$\sigma-\sigma$	D	0.0525	8	Methane dimer	$\sigma-\sigma$	D	0.0106
Hard_GW	1	Ammonia dimer	HB	E	0.0448	8	Methane dimer	$\sigma-\sigma$	D	0.0106
Standard	14	Indole-benzene(stack)	$\pi-\pi$	D	0.0277	9	Ethene dimer	$\pi-\pi$	D	0.0153
Standard_GW	14	Indole-benzene(stack)	$\pi-\pi$	D	0.0176	8	Methane dimer	$\sigma-\sigma$	D	0.0232
Soft	11	Benzene dimer(stack)	$\pi-\pi$	D	0.0119	20	Benzene dimer(TS)	others	D	0.0467
AVDZ	8	Methane dimer	$\sigma-\sigma$	D	0.2495	11	Benzene dimer(stack)	$\pi-\pi$	D	0.0286
AVTZ	12	Pyrazine dimer	$\pi-\pi$	D	0.0664	8	Methane dimer	$\sigma-\sigma$	D	0.0356
AVQZ	22	Phenol dimer	others	M	0.0014	9	Ethene dimer	$\pi-\pi$	D	0.0035

TABLE ST19: The minimum absolute error of Hard, Hard_GW, Standard, Standard_GW and Soft as well as AVDZ, AVTZ and AVQZ for HF method and PBE functionl on S66 database. The errors are in meV.

	PBE					HF				
potential/basis set	number	complex	type	nature	min	number	complex	type	nature	min
Hard	5	MeOH-MeOH	HB	E	0.0038	21	AcNH2-AcNH2	HB	E	0.0083
Hard_GW	7	MeOH-Peptide	HB	E	0.0034	31	Uracil-Ethene	$\pi-\pi$	D	0.0022
Standard	36	Neopentane-Neopentane	$\sigma-\sigma$	D	0.0085	35	Neopentane-Pentane	$\sigma-\sigma$	D	0.0076
Standard_GW	34	Pentane-Pentane	$\sigma-\sigma$	D	0.0221	51	Ethyne-Ethyne(TS)	others	M	0.0295
Soft	24	Benzene-Benzene(π-π)	$\pi-\pi$	D	0.0187	38	Cyclopentane-Cyclopentane	$\sigma-\sigma$	D	0.0037
AVDZ	38	Cyclopentane-Cyclopentane	$\sigma-\sigma$	D	0.0016	3	Water-MeNH2	HB	E	0.0033
AVTZ	25	Pyridine-Pyridine(π-π)	$\pi-\pi$	D	0.0027	47	Benzene-Benzene(TS)	others	D	0.0019
AVQZ	46	Peptide-Pentane	$\pi-\sigma$	D	0.0033	63	Benzene-AcOH	others	D	0.0

TABLE ST20: The coefficients of the fitting ($\mathrm{E_{int}^{err}} = \mathrm{ae^{-bR}} + \mathrm{cR^{-3}}$) equation of Hard potential error for the hydrogen bonded sample complexes.

Complex	a	b	c
$H_2O\cdots CH_4$	-245.47	3.32	0.23
$H_2O\cdots CO_2$	-391.88	3.97	0.93
$H_2O\cdots CO$	-1430.78	5.35	-1.55
$H_2O\cdots NH_3$	-183.25	3.02	4.08
$H_2O\cdots H_2O$	-129.03	2.72	6.45

TABLE ST21: The coefficients of the fitting ($\mathrm{E_{int}^{err}} = \mathrm{ae^{-bR}} + \mathrm{cR^{-3}}$) equation of Hard_*GW* potential error for the hydrogen bonded sample complexes.

Complex	a	b	c
$H_2O\cdots CH_4$	6.67	0.94	-10.45
$H_2O\cdots CO_2$	-328.38	4.10	4.97
$H_2O\cdots CO$	5.64	1.44	-1.05
$H_2O\cdots NH_3$	-131.80	2.88	4.56
$H_2O\cdots H_2O$	-99.97	2.55	9.17

TABLE ST22: The coefficients of the fitting ($E_{int}^{err} = ae^{-bR} + cR^{-3}$) equation of Standard potential error for the hydrogen bonded sample complexes.

Complex	a	b	c
$H_2O\cdots CH_4$	−1092.56	3.64	−8.00
$H_2O\cdots CO_2$	−386.52	3.06	−33.11
$H_2O\cdots CO$	−441.84	3.50	−40.51
$H_2O\cdots NH_3$	−150.00	3.07	−24.03
$H_2O\cdots H_2O$	−5.58	1.14	−44.79

TABLE ST23: The coefficients of the fitting ($E_{int}^{err} = ae^{-bR} + cR^{-3}$) equation of Standard_*GW* potential error for the hydrogen bonded sample complexes.

Complex	a	b	c
$H_2O\cdots CH_4$	−1152.96	3.55	−6.65
$H_2O\cdots CO_2$	−821.91	3.32	−27.14
$H_2O\cdots CO$	20.30	0.98	−66.22
$H_2O\cdots NH_3$	−503.26	3.30	−19.33
$H_2O\cdots H_2O$	−264.79	2.97	−38.98

TABLE ST24: The coefficients of the fitting ($E_{int}^{err} = ae^{-bR} + cR^{-3}$) equation of Soft potential error for the hydrogen bonded sample complexes.

Complex	a	b	c
$H_2O\cdots CH_4$	40.03	0.92	−92.52
$H_2O\cdots CO_2$	17.41	0.80	−171.42
$H_2O\cdots CO$	7.52	0.72	−169.90
$H_2O\cdots NH_3$	2.94	0.68	−99.56
$H_2O\cdots H_2O$	−14.38	0.94	−143.84

TABLE ST25: The coefficients of the fitting ($E_{int}^{err} = ae^{-bR} + cR^{-3}$) equation of Hard potential error for the direct oxygen-oxygen contact sample complexes.

Complex	a	b	c
$CO_2 \cdots CO$	−4063.77	3.45	1.57
$CO_2 \cdots CO_2$	−4112.76	3.41	1.32
$H_2O \cdots CO_2$	−3538.51	3.33	−0.06
$H_2O \cdots CO$	−3630.17	3.39	2.94
$H_2O \cdots H_2O$	−2649.91	3.23	−10.33
$O_2 \cdots O_2$	−3087.93	3.37	1.23

TABLE ST26: The coefficients of the fitting ($E_{int}^{err} = ae^{-bR} + cR^{-3}$) equation of Hard_*GW* potential error for the direct oxygen-oxygen contact sample complexes.

Complex	a	b	c
$CO_2 \cdots CO$	−2192.87	3.21	−1.04
$CO_2 \cdots CO_2$	−2344.32	3.19	−3.36
$H_2O \cdots CO_2$	−1891.88	3.09	−6.96
$H_2O \cdots CO$	−1730.45	3.10	−1.24
$H_2O \cdots H_2O$	−1107.05	2.70	1.08
$O_2 \cdots O_2$	−1995.09	3.21	0.91

TABLE ST27: The coefficients of the fitting ($E_{int}^{err} = ae^{-bR} + cR^{-3}$) equation of Standard potential error for the direct oxygen-oxygen contact sample complexes.

Complex	a	b	c
$CO_2 \cdots CO$	−203841.91	5.48	36.17
$CO_2 \cdots CO_2$	−131753.53	5.03	35.08
$H_2O \cdots CO_2$	−105168.45	4.83	60.44
$H_2O \cdots CO$	−196667.39	5.39	71.21
$H_2O \cdots H_2O$	−69180.26	4.50	91.71
$O_2 \cdots O_2$	−44016.37	4.30	4.07

TABLE ST28: The coefficients of the fitting ($E_{int}^{err} = ae^{-bR} + cR^{-3}$) equation of Standard_GW potential error for the direct oxygen-oxygen contact sample complexes.

Complex	a	b	c
$CO_2 \cdots CO$	−167653.53	5.42	29.89
$CO_2 \cdots CO_2$	−110071.73	4.98	26.59
$H_2O \cdots CO_2$	−92807.39	4.79	47.98
$H_2O \cdots CO$	−163218.00	5.31	64.18
$H_2O \cdots H_2O$	−65874.95	4.50	72.86
$O_2 \cdots O_2$	−47341.93	4.41	1.40

TABLE ST29: The coefficients of the fitting ($E_{int}^{err} = ae^{-bR} + cR^{-3}$) equation of Soft potential error for the direct oxygen-oxygen contact sample complexes.

Complex	a	b	c
$CO_2 \cdots CO$	−109566173.91	9.48	187.2
$CO_2 \cdots CO_2$	−3416034.77	6.91	155.89
$H_2O \cdots CO_2$	−801669.26	5.73	225.17
$H_2O \cdots CO$	−5354382.88	7.13	273.13
$H_2O \cdots H_2O$	−233433.44	4.74	300.46
$O_2 \cdots O_2$	−388090.00	5.27	21.55

TABLE ST30: The PBE interaction energies for S66 datasets for Hard, Hard_GW, Standard, Standard_GW, Soft, pslibrary1.0.0 (precision), pslibrary1.0.0 (efficiency) potentials as well as AVDZ, AVTZ, AVQZ and AV5Z basis sets. The Energies are in eV.

Dimer number	Hard	Hard_GW	Standard	Standard_GW	Soft	precision	efficiency	AVDZ	AVTZ	AVQZ	AV5Z
1	−0.21101	−0.21086	−0.21658	−0.21613	−0.22888	−0.214008	−0.215498	−0.208373	−0.209322	−0.210952	−0.211067
2	−0.22358	−0.22350	−0.22969	−0.22938	−0.24350	−0.226891	−0.228525	−0.222182	−0.221777	−0.223548	−0.223693
3	−0.30853	−0.30877	−0.31388	−0.31380	−0.32873	−0.312267	−0.313288	−0.307512	−0.307685	−0.308473	−0.308493
4	−0.31628	−0.31611	−0.32311	−0.32308	−0.34133	−0.320541	−0.322558	−0.312387	−0.314327	−0.316231	−0.316373
5	−0.22099	−0.22084	−0.22724	−0.22684	−0.24076	−0.224299	−0.225982	−0.218588	−0.219054	−0.220822	−0.220989
6	−0.31329	−0.31349	−0.31852	−0.31841	−0.33265	−0.316970	−0.317930	−0.311819	−0.312539	−0.313109	−0.313196
7	−0.31262	−0.31245	−0.31886	−0.31883	−0.33511	−0.316237	−0.318084	−0.307687	−0.310414	−0.312371	−0.312452
8	−0.20823	−0.20800	−0.21397	−0.21342	−0.22615	−0.211247	−0.212792	−0.204719	−0.206439	−0.208050	−0.208198
9	−0.09807	−0.09812	−0.09998	−0.09978	−0.10466	−0.099605	−0.099989	−0.097307	−0.097425	−0.097983	−0.098101
10	−0.13467	−0.13497	−0.13626	−0.13612	−0.14161	−0.136639	−0.136639	−0.133535	−0.134293	−0.134744	−0.134828
11	−0.14882	−0.14898	−0.15089	−0.15087	−0.15773	−0.151123	−0.151525	−0.147493	−0.148400	−0.148999	−0.149065
12	−0.30956	−0.30982	−0.31525	−0.31517	−0.33106	−0.313565	−0.314651	−0.308646	−0.308737	−0.309475	−0.309621
13	−0.19908	−0.19893	−0.20412	−0.20361	−0.21588	−0.201738	−0.203132	−0.195090	−0.197163	−0.198332	−0.198425
14	−0.26786	−0.26816	−0.27023	−0.27009	−0.27887	−0.270045	−0.270283	−0.266341	−0.266483	−0.267136	−0.267222
15	−0.27976	−0.27967	−0.28337	−0.28333	−0.29494	−0.281769	−0.282935	−0.275887	−0.277070	−0.278510	−0.278517
16	−0.18940	−0.18917	−0.19400	−0.19342	−0.20468	−0.191699	−0.192971	−0.185198	−0.187555	−0.188508	−0.188641
17	−0.67802	−0.67724	−0.68743	−0.68756	−0.72104	−0.685606	−0.689017	−0.670221	−0.673086	−0.676963	−0.677068
18	−0.29535	−0.29554	−0.30088	−0.30083	−0.31709	−0.299360	−0.300330	−0.294477	−0.294119	−0.295073	−0.295195
19	−0.30204	−0.30217	−0.30758	−0.30748	−0.32345	−0.306073	−0.307013	−0.300380	−0.300708	−0.301653	−0.301803
20	−0.81144	−0.81047	−0.82500	−0.82627	−0.86820	−0.820913	−0.825513	−0.799717	−0.807161	−0.812015	−0.812322
21	−0.65369	−0.65318	−0.66153	−0.66177	−0.68969	−0.661050	−0.663939	−0.649989	−0.650515	−0.654688	−0.654778
22	−0.79917	−0.79824	−0.81098	−0.81169	−0.85023	−0.808589	−0.812773	−0.789338	−0.795279	−0.799528	−0.799723
23	−0.77027	−0.76951	−0.77956	−0.77987	−0.81287	−0.778344	−0.781824	−0.764310	−0.766504	−0.770337	−0.770439
24	0.07763	0.07760	0.07768	0.07769	0.07778	0.077034	0.077034	0.076096	0.077810	0.077704	0.077796
25	0.04208	0.04199	0.04182	0.04179	0.04068	0.041182	0.041182	0.040378	0.042130	0.041971	0.042132
26	−0.11829	−0.11837	−0.11972	−0.11985	−0.12574	−0.120265	−0.120814	−0.116087	−0.118919	−0.119025	−0.118633
27	0.05650	0.05645	0.05638	0.05637	0.05576	0.055762	0.055762	0.054803	0.056684	0.056563	0.056664
28	0.02065	0.02065	0.01978	0.01982	0.01703	0.019366	0.019048	0.020722	0.020804	0.020719	0.020897
29	−0.02887	−0.02893	−0.03019	−0.03018	−0.03474	−0.031063	−0.031413	−0.029342	−0.029317	−0.029475	−0.029212
30	0.03951	0.03952	0.03955	0.03957	0.03960	0.039098	0.039098	0.038611	0.039749	0.039455	0.039650
31	−0.01954	−0.01953	−0.01981	−0.01985	−0.02092	−0.020076	−0.020207	−0.018826	−0.019192	−0.019422	−0.019344
32	−0.04823	−0.04823	−0.04875	−0.04882	−0.05045	−0.048955	−0.049183	−0.047124	−0.047974	−0.048241	−0.048006
33	0.02314	0.02314	0.02306	0.02309	0.02267	0.022603	0.022603	0.022422	0.023383	0.023052	0.023265
34	0.01194	0.01186	0.01198	0.01191	0.01202	0.011463	0.011463	0.010079	0.012646	0.011923	0.011928
35	0.00134	0.00128	0.00135	0.00129	0.00135	0.000966	0.000966	0.002945	0.002251	0.001539	0.001525
36	−0.00249	−0.00252	−0.00246	−0.00250	−0.00236	−0.002792	−0.002792	−0.002427	−0.001923	−0.002463	−0.002450
37	0.00743	0.00737	0.00744	0.00737	0.00747	0.007053	0.007053	0.008204	0.008153	0.007465	0.007533
38	0.00218	0.00212	0.00220	0.00213	0.00223	0.001759	0.001759	0.002298	0.003013	0.002263	0.002299
39	0.01633	0.01626	0.01639	0.01632	0.01649	0.015895	0.015895	0.015078	0.016740	0.016451	0.016545
40	0.00447	0.00441	0.00452	0.00445	0.00460	0.004124	0.004124	0.005572	0.004773	0.004513	0.004691

Continued

TABLE ST30 − *continued*

Dimer number	Hard	Hard_GW	Standard	Standard_GW	Soft	precision	efficiency	AVDZ	AVTZ	AVQZ	AV5Z
41	0.01271	0.01263	0.01274	0.01263	0.01234	0.012242	0.012222	0.013609	0.013328	0.012797	0.012930
42	0.01626	0.01620	0.01634	0.01625	0.01620	0.015861	0.015857	0.017285	0.016999	0.016518	0.016588
43	−0.00597	−0.00600	−0.00607	−0.00611	−0.00671	−0.006453	−0.006502	−0.005966	−0.005582	−0.005780	−0.005742
44	−0.00680	−0.00685	−0.00679	−0.00683	−0.00683	−0.007058	−0.007058	−0.007842	−0.006261	−0.006665	−0.006720
45	−0.00540	−0.00545	−0.00538	−0.00541	−0.00537	−0.005636	−0.005636	−0.006901	−0.005195	−0.005340	−0.005273
46	−0.00918	−0.00927	−0.00932	−0.00940	−0.01002	−0.009715	−0.009771	−0.009101	−0.008581	−0.009089	−0.009092
47	−0.00325	−0.00334	−0.00318	−0.00329	−0.00311	−0.003467	−0.003467	−0.002302	−0.002845	−0.003145	−0.002966
48	−0.03033	−0.03048	−0.03053	−0.03066	−0.03150	−0.030435	−0.030435	−0.029519	−0.029724	−0.030051	−0.029800
49	−0.02274	−0.02286	−0.02279	−0.02291	−0.02312	−0.022943	−0.022943	−0.021471	−0.022345	−0.022596	−0.022403
50	−0.05278	−0.05293	−0.05263	−0.05285	−0.05217	−0.052644	−0.052644	−0.048785	−0.052664	−0.052587	−0.052380
51	−0.05392	−0.05403	−0.05380	−0.05398	−0.05326	−0.053763	−0.053763	−0.052110	−0.053610	−0.053788	−0.053677
52	−0.10397	−0.10402	−0.10513	−0.10534	−0.10855	−0.104664	−0.105046	−0.097582	−0.103210	−0.103630	−0.103788
53	−0.10285	−0.10296	−0.10358	−0.10375	−0.10652	−0.103538	−0.103805	−0.100130	−0.102233	−0.102707	−0.102711
54	−0.08766	−0.08772	−0.08893	−0.08899	−0.09191	−0.088353	−0.088707	−0.085404	−0.087116	−0.087401	−0.087487
55	−0.08273	−0.08279	−0.08390	−0.08394	−0.08634	−0.083405	−0.083735	−0.078730	−0.082125	−0.082463	−0.082450
56	−0.03569	−0.03580	−0.03590	−0.03596	−0.03672	−0.036207	−0.036207	−0.032932	−0.035299	−0.035612	−0.035495
57	−0.07160	−0.07172	−0.07194	−0.07207	−0.07325	−0.071962	−0.072096	−0.067441	−0.071097	−0.071313	−0.071144
58	−0.10839	−0.10871	−0.10974	−0.10978	−0.11462	−0.110062	−0.110062	−0.109311	−0.108344	−0.108571	−0.108647
59	−0.11456	−0.11454	−0.11690	−0.11678	−0.12156	−0.115681	−0.116347	−0.112054	−0.113798	−0.114247	−0.114310
60	−0.18123	−0.18127	−0.18298	−0.18341	−0.18805	−0.182136	−0.182784	−0.174971	−0.180231	−0.180870	−0.181045
61	−0.00859	−0.00864	−0.00863	−0.00870	−0.00905	−0.008942	−0.008965	−0.008400	−0.007910	−0.008276	−0.008393
62	−0.02236	−0.02243	−0.02258	−0.02266	−0.02353	−0.022921	−0.022998	−0.021779	−0.021728	−0.022315	−0.022342
63	−0.02418	−0.02425	−0.02459	−0.02471	−0.02607	−0.024787	−0.024962	−0.023877	−0.023707	−0.023957	−0.023869
64	−0.04633	−0.04640	−0.04671	−0.04678	−0.04816	−0.046785	−0.046919	−0.045432	−0.045844	−0.046221	−0.046146
65	−0.15917	−0.15949	−0.16018	−0.16039	−0.16339	−0.160044	−0.160044	−0.158907	−0.158513	−0.158815	−0.158821
66	−0.07686	−0.07705	−0.07790	−0.07787	−0.08168	−0.078285	−0.078285	−0.076596	−0.076413	−0.076872	−0.076794

TABLE ST31: The HF interaction energies for S66 datasets for Hard, Hard_GW, Standard, Standard_GW, Soft, as well as AVDZ, AVTZ, AVQZ and AV5Z basis sets. The Energies are in eV.

Dimer number	Hard	Hard_GW	Standard	Standard_GW	Soft	AVDZ	AVTZ	AVQZ	AV5Z
1	−0.163757	−0.163012	−0.169338	−0.168436	−0.180056	−0.162472	−0.161548	−0.163030	−0.163015
2	−0.167091	−0.166405	−0.172828	−0.172037	−0.184441	−0.166480	−0.164594	−0.166255	−0.166207
3	−0.207853	−0.207188	−0.211791	−0.211333	−0.223812	−0.206627	−0.206038	−0.206637	−0.206630
4	−0.257462	−0.256692	−0.264450	−0.263897	−0.280194	−0.253975	−0.255095	−0.256512	−0.256517
5	−0.158564	−0.157413	−0.164403	−0.163543	−0.175352	−0.156790	−0.155905	−0.157688	−0.157701
6	−0.191831	−0.191270	−0.195482	−0.195059	−0.206371	−0.190318	−0.189923	−0.190638	−0.190688
7	−0.232177	−0.231490	−0.238137	−0.237725	−0.250882	−0.228355	−0.229264	−0.231135	−0.231207
8	−0.155875	−0.154698	−0.161597	−0.160621	−0.171842	−0.153509	−0.153558	−0.155107	−0.155141
9	−0.047973	−0.047751	−0.049490	−0.049395	−0.053847	−0.048472	−0.046926	−0.047631	−0.047644
10	−0.046865	−0.046577	−0.047345	−0.047510	−0.052433	−0.045623	−0.045822	−0.046313	−0.046321
11	−0.073387	−0.073079	−0.074934	−0.075254	−0.081721	−0.072934	−0.072183	−0.072954	−0.073000
12	−0.203840	−0.203102	−0.208027	−0.207575	−0.220934	−0.202534	−0.202069	−0.202568	−0.202568
13	−0.143151	−0.142392	−0.147491	−0.147016	−0.158364	−0.139912	−0.140317	−0.141318	−0.141332
14	−0.156144	−0.155660	−0.156719	−0.157002	−0.165087	−0.155450	−0.154011	−0.154481	−0.154503
15	−0.207317	−0.206769	−0.210200	−0.210455	−0.220456	−0.204166	−0.203912	−0.205262	−0.205293
16	−0.153381	−0.152439	−0.157843	−0.157172	−0.168093	−0.150174	−0.150852	−0.151678	−0.151710
17	−0.563461	−0.560924	−0.573467	−0.573632	−0.605795	−0.551410	−0.556892	−0.560416	−0.560506
18	−0.199532	−0.198944	−0.203880	−0.203462	−0.216994	−0.195376	−0.197655	−0.198304	−0.198324
19	−0.189495	−0.188823	−0.193743	−0.193314	−0.206064	−0.184947	−0.187584	−0.188300	−0.188366
20	−0.680301	−0.678670	−0.693734	−0.694110	−0.726510	−0.662113	−0.674784	−0.678998	−0.679349
21	−0.538267	−0.537026	−0.545343	−0.545871	−0.570187	−0.533079	−0.534494	−0.538182	−0.538259
22	−0.685575	−0.683478	−0.697837	−0.698016	−0.731786	−0.670388	−0.680332	−0.684008	−0.684235
23	−0.667594	−0.666364	−0.676966	−0.677437	−0.708372	−0.658775	−0.662725	−0.665997	−0.666122
24	0.226743	0.226857	0.226911	0.226689	0.226371	0.227735	0.227383	0.227276	0.227216
25	0.192075	0.192500	0.192209	0.191791	0.190445	0.192272	0.192818	0.192684	0.192631
26	0.001705	0.002171	0.001535	0.000361	−0.002853	0.002432	0.002198	0.001909	0.001829
27	0.204721	0.205708	0.204855	0.204538	0.203664	0.205191	0.205442	0.205335	0.205279
28	0.163654	0.163957	0.162811	0.162518	0.159663	0.165446	0.164485	0.164477	0.164448
29	0.101315	0.101968	0.100098	0.099621	0.095282	0.103187	0.101555	0.101501	0.101457
30	0.145328	0.145632	0.145242	0.145178	0.144818	0.146823	0.145694	0.145547	0.145508
31	0.054764	0.055167	0.054467	0.054253	0.053132	0.057718	0.055269	0.055191	0.055164
32	0.012796	0.013539	0.012218	0.012029	0.010291	0.016326	0.013183	0.013264	0.013250
33	0.129161	0.129543	0.128999	0.128927	0.128163	0.130703	0.129592	0.129408	0.129389
34	0.137097	0.137451	0.137521	0.137276	0.137546	0.137798	0.137682	0.137529	0.137480
35	0.092805	0.092848	0.093136	0.092937	0.093138	0.094397	0.093322	0.093170	0.093129
36	0.063568	0.063699	0.063813	0.063671	0.063976	0.064501	0.063864	0.063792	0.063758
37	0.100883	0.101293	0.101246	0.101028	0.101274	0.102147	0.101419	0.101278	0.101230
38	0.108386	0.108703	0.108733	0.108529	0.108721	0.109268	0.108902	0.108774	0.108725
39	0.135974	0.136066	0.136412	0.136141	0.136222	0.136844	0.136691	0.136520	0.136499
40	0.096819	0.097048	0.097258	0.096991	0.097101	0.097995	0.097478	0.097333	0.097323
41	0.133848	0.134464	0.134451	0.133912	0.134211	0.135603	0.134553	0.134447	0.134370

Continued

TABLE ST31 – *continued*

Dimer number	Hard	Hard_GW	Standard	Standard_GW	Soft	AVDZ	AVTZ	AVQZ	AV5Z
42	0.124248	0.124702	0.124860	0.124386	0.124682	0.125632	0.124860	0.124729	0.124672
43	0.074253	0.074638	0.074416	0.074127	0.073752	0.075712	0.074737	0.074663	0.074613
44	0.063620	0.063859	0.063851	0.063716	0.063756	0.063997	0.063993	0.063887	0.063862
45	0.062814	0.062973	0.063004	0.062887	0.062783	0.062792	0.063182	0.063115	0.063093
46	0.100162	0.100202	0.100512	0.100117	0.099924	0.101663	0.100778	0.100597	0.100533
47	0.074663	0.074868	0.073359	0.074992	0.075270	0.074388	0.075303	0.075284	0.075305
48	0.042626	0.043060	0.043385	0.042823	0.042425	0.041269	0.043701	0.043608	0.043615
49	0.050096	0.049835	0.050812	0.050367	0.050421	0.049855	0.050816	0.050823	0.050846
50	−0.006037	−0.005813	−0.004877	−0.005392	−0.004360	−0.004279	−0.005582	−0.005339	−0.005281
51	−0.024466	−0.024264	−0.023723	−0.024015	−0.023307	−0.022313	−0.023948	−0.024073	−0.024044
52	−0.035697	−0.034314	−0.035885	−0.036329	−0.038051	−0.032119	−0.035028	−0.034886	−0.034888
53	−0.043397	−0.043140	−0.043204	−0.043688	−0.045665	−0.045427	−0.042806	−0.042715	−0.042743
54	−0.040990	−0.040667	−0.041768	−0.041916	−0.044186	−0.041979	−0.040211	−0.040320	−0.040304
55	0.002697	0.003233	0.002280	0.002093	0.000669	0.003739	0.003520	0.003458	0.003451
56	0.045488	0.045740	0.045833	0.045539	0.045010	0.046566	0.046035	0.045983	0.045997
57	0.027137	0.027586	0.027817	0.027303	0.026784	0.028403	0.028056	0.028138	0.028145
58	−0.051481	−0.051250	−0.051466	−0.051912	−0.056204	−0.051434	−0.050891	−0.051013	−0.051026
59	−0.097998	−0.097761	−0.100032	−0.099830	−0.104853	−0.096508	−0.096680	−0.097139	−0.097179
60	−0.119020	−0.118131	−0.119858	−0.120349	−0.123456	−0.112053	−0.118226	−0.118231	−0.118377
61	0.068522	0.068732	0.068827	0.068517	0.068506	0.069294	0.069006	0.068923	0.068864
62	0.071327	0.071366	0.071498	0.071120	0.070641	0.072386	0.071830	0.071700	0.071630
63	0.051737	0.052721	0.051733	0.051301	0.050138	0.051239	0.052523	0.052357	0.052357
64	0.013577	0.013703	0.013532	0.013235	0.012000	0.013571	0.014190	0.014016	0.013996
65	−0.112194	−0.111894	−0.111833	−0.112231	−0.115264	−0.110944	−0.111055	−0.111326	−0.111300
66	0.014862	0.014834	0.014645	0.014291	0.011145	0.014485	0.015746	0.015289	0.015274

TABLE ST32: The PBE interaction energies for S22 datasets for Hard, Hard_GW, Standard, Standard_GW, Soft, as well as AVDZ, AVTZ, AVQZ and AV5Z basis sets. The Energies are in eV.

Number	Dimer	Hard	Hard_GW	Standard	Standard_GW	Soft	AVDZ	AVTZ	AVQZ	AV5Z
1	Ammonia dimer	−0.121656	−0.121912	−0.123149	−0.122970	−0.128118	−0.118748	−0.121715	−0.121836	−0.121867
2	Water dimer	−0.214454	−0.214327	−0.220149	−0.219822	−0.232852	−0.211885	−0.212694	−0.214412	−0.214507
3	Formic acid dimer	−0.790930	−0.789841	−0.804632	−0.806164	−0.848362	−0.776479	−0.786704	−0.791620	−0.791914
4	Formamide dimer	−0.640415	−0.639848	−0.648320	−0.648702	−0.676884	−0.634519	−0.637386	−0.641558	−0.641655
5	Uracil dimer	−0.804017	−0.803039	−0.814142	−0.814429	−0.850246	−0.797176	−0.801779	−0.805385	−0.805502
6	2-pyridoxine-2-aminopyridine	−0.666360	−0.666430	−0.672557	−0.672820	−0.697760	−0.662568	−0.664360	−0.667013	−0.667206
7	Adenine-thymine	−0.622863	−0.622789	−0.629324	−0.629523	−0.655779	−0.619286	−0.620921	−0.623795	−0.623993
8	Methane dimer	−0.004237	−0.004237	−0.004217	−0.004222	−0.004161	−0.004434	−0.004398	−0.004134	−0.004184
9	Ethene dimer	−0.014310	−0.014365	−0.014303	−0.014357	−0.014400	−0.014495	−0.013844	−0.014211	−0.014190
10	Benzene-methane	−0.002243	−0.002277	−0.002205	−0.002253	−0.002146	−0.000130	−0.001785	−0.002263	−0.002083
11	Benzene dimer(stack)	0.080098	0.080062	0.080152	0.080154	0.080249	0.078170	0.080335	0.080219	0.080261
12	Pyrazine dimer	0.029570	0.029474	0.029122	0.029064	0.027166	0.029317	0.029858	0.029495	0.029792
13	Uracil dimer(stack)	−0.118290	−0.118361	−0.119719	−0.119842	−0.125740	−0.116082	−0.118915	−0.119021	−0.118629
14	Indole-benzene(stack)	0.094231	0.094185	0.094331	0.094341	0.094501	0.090811	0.094482	0.094347	0.094358
15	Adenine-thymine(stack)	−0.061065	−0.061104	−0.062741	−0.062853	−0.068924	−0.062766	−0.061194	−0.061537	−0.061315
16	Ethene-ethyne	−0.051161	−0.051270	−0.051081	−0.051241	−0.050786	−0.049234	−0.051045	−0.051022	−0.050911
17	Benzene-water	−0.087767	−0.087818	−0.089031	−0.089091	−0.092005	−0.085534	−0.087223	−0.087504	−0.087589
18	Benzene-ammonia	−0.040642	−0.040746	−0.040864	−0.040936	−0.041762	−0.037955	−0.040114	−0.040547	−0.040497
19	Benzene-HCN	−0.122926	−0.123149	−0.123052	−0.123511	−0.122694	−0.116029	−0.123020	−0.122693	−0.122383
20	Benzene dimer(TS)	−0.005757	−0.005847	−0.005682	−0.005791	−0.005589	−0.002723	−0.005282	−0.005603	−0.005494
21	Indolebenzene(TS)	−0.089574	−0.089758	−0.089391	−0.089547	−0.089305	−0.082333	−0.088863	−0.089136	−0.089023
22	Phenol dimer	−0.169306	−0.169116	−0.176131	−0.175762	−0.191136	−0.165750	−0.167214	−0.168954	−0.168955

TABLE ST33: The HF interaction energies for S22 datasets for Hard, Hard_GW, Standard, Standard_GW, Soft, as well as AVDZ, AVTZ, AVQZ and AV5Z basis sets. The Energies are in eV.

Number	Dimer	Hard	Hard_GW	Standard	Standard_GW	Soft	AVDZ	AVTZ	AVQZ	AV5Z
1	Ammonia dimer	−0.061278	−0.061100	−0.061748	−0.061894	−0.066212	−0.059460	−0.060714	−0.060985	−0.061011
2	Water dimer	−0.156188	−0.155477	−0.161714	−0.160909	−0.172366	−0.154740	−0.153889	−0.155474	−0.155452
3	Formic acid dimer	−0.663944	−0.661868	−0.677152	−0.677914	−0.708841	−0.642954	−0.658663	−0.662789	−0.663199
4	Formamide dimer	−0.528341	−0.528258	−0.535190	−0.535991	−0.559358	−0.522010	−0.524739	−0.528429	−0.528560
5	Uracil dimer	−0.705744	−0.703705	−0.717012	−0.717084	−0.752893	−0.695054	−0.702556	−0.705666	−0.705817
6	2-pyridoxine-2-aminopyridine	−0.455683	−0.452547	−0.459387	−0.460422	−0.483866	−0.446956	−0.452228	−0.454435	−0.454568
7	Adenine-thymine	−0.436877	−0.435546	−0.440750	−0.442095	−0.466450	−0.429379	−0.434066	−0.436219	−0.436368
8	Methane dimer	0.015881	0.015902	0.015945	0.015915	0.016061	0.015616	0.015927	0.015910	0.015892
9	Ethene dimer	0.035545	0.035651	0.035911	0.035727	0.035780	0.035979	0.035950	0.035899	0.035895
10	Benzene-methane	0.050414	0.050437	0.050689	0.050524	0.050628	0.051357	0.050857	0.050690	0.050705
11	Benzene dimer(stack)	0.231653	0.232153	0.231874	0.231629	0.231352	0.232220	0.232343	0.232254	0.232191
12	Pyrazine dimer	0.179834	0.180307	0.180025	0.179354	0.177534	0.181004	0.180834	0.180581	0.180520
13	Uracil dimer(stack)	0.001709	0.002173	0.001539	0.000365	0.002911	0.002435	0.002202	0.001912	0.001832
14	Indole-benzene(stack)	0.304464	0.304962	0.304798	0.304488	0.304277	0.306043	0.305299	0.305264	0.305189
15	Adenine-thymine(stack)	0.142068	0.142783	0.141571	0.140243	0.136567	0.143806	0.142830	0.142634	0.142539
16	Ethene-ethyne	−0.019174	−0.019168	−0.018485	−0.018746	−0.018289	−0.018001	−0.018786	−0.018746	−0.018696
17	Benzene-water	−0.041368	−0.040983	−0.042146	−0.042292	−0.044562	−0.042389	−0.040592	−0.040701	−0.040685
18	Benzene-ammonia	0.007329	0.007496	0.007571	0.007302	0.006601	0.007975	0.007898	0.007726	0.007757
19	Benzene-HCN	−0.087362	−0.087454	−0.085787	−0.086974	−0.085759	−0.084578	−0.086709	−0.086379	−0.086318
20	Benzene dimer(TS)	0.065106	0.065455	0.065797	0.065447	0.065785	0.065698	0.065603	0.065717	0.065738
21	Indolebenzene(TS)	0.011303	0.011684	0.013100	0.012439	0.014131	0.013730	0.012103	0.012469	0.012487
22	Phenol dimer	−0.081403	−0.080484	−0.087277	−0.086633	−0.099534	−0.078839	−0.078347	−0.079767	−0.079815
